# Critically Exploring Self‐Harm Through Lived Experience Perspectives: A Survivor‐Controlled Integrative Review Employing Participatory Methodology

**DOI:** 10.1111/hex.70700

**Published:** 2026-06-07

**Authors:** C. C. da Cunha Lewin, M. Leamy, S Baldoza, G. Jerwood, Es Miles, U. Foye, A. Sweeney

**Affiliations:** ^1^ Health Service and Population Research, Institute of Psychology, Psychiatry and Neuroscience (IoPPN) King's College London London UK; ^2^ Lived Experience Advisory Panel (LEAP), Service User Research Enterprise, Health Service and Population Research, Institute of Psychology, Psychiatry and Neuroscience (IoPPN) King's College London London UK; ^3^ Florence Nightingale Faculty of Nursing, Midwifery and Palliative Care, King's College London London UK

**Keywords:** integrative review, lived experience, self‐harm, survivor approach

## Abstract

**Introduction:**

In psychiatric knowledge, self‐harm is commonly understood as an individual, self‐destructive behaviour arising from emotional dysregulation where sociocultural context is referred to briefly but typically overlooked. Conversely, many with lived experience (LE) highlight relational and systemic trauma, abuse and violence as inseparable from self‐harm. This survivor‐controlled integrative review was led by a LE researcher and employed participatory methodology with people with LE to explore self‐harm. Our research question was *‘How is self‐harm conceptualised?’*.

**Methods:**

Evidence was identified from a systematic search of MEDLINE, EMBASE, PsycINFO, CINAHL and Global Health. Eligible studies must have explored self‐harm subjectivity, used qualitative methodology and PPI. PPI was assessed based on NIHR ‘Levels of Involvement’. 36 articles were appraised using CASP, GRIPP‐2 and an adapted Equity, Diversity and Inclusion framework. Reflexive thematic analysis within social constructionism and a survivor approach were employed.

**Results:**

Findings were organised into two themes. **Theme 1**: **What is self‐harm?** describes how researchers variably constructed self‐harm across the included studies. **Theme 2**: **The web of self‐harm** explores its ‘*trajectory’*, exploring how social experiences and circumstances work to induce chaotic embodied sensations, leading to self‐harm as an emotional practice to negotiate with oneself and one's experiences. As an embodied practice, self‐harm reflects the liminality between the personal/social and speaks to what it means to exist in social life.

**Conclusion:**

This review explores how self‐harm can be constructed as an embodied practice mediated by social circumstances, highlighting how psy‐knowledge overlooks social life. In identifying this, we highlight how common clinical responses that individualise and pathologise LE are rationalised. Through PPI and survivor methods, this work offers significant methodological contribution, demonstrating how LE inclusion supported critical, interdisciplinary understanding, enhancing knowledge to reconnect people to their experiences.

**Patient or Public Contribution:**

This was a survivor‐controlled research project. The lead author, CdCL, is a LE researcher, and general nurse, who centralises experiences of complex trauma, mental distress and self‐harm in their work. AS is also a survivor researcher with extensive experience in trauma‐informed approaches. UF is a LE researcher with significant expertise in eating distress and qualitative methods. CdCL collaborated with a PPI advisory board throughout the research project to ensure greater inclusivity and transferability to others with LE. PPI peers SB, GJ and EM, are co‐authors to this work, and supported the refinement of research objectives, interpretation, naming and development of themes and edited the manuscript.

## 1 Introduction

Within psychiatric knowledge [1], mental ‘illness’, or mental distress [1] is compartmentalised into psychopathological behaviours that require containment to achieve clinical ‘recovery’, as aligned with the biomedical model [2, 3]. Likewise, self‐harm is seen as a destructive behaviour [4], that needs to stop [5, 6]. This understanding is influenced by the sociological and psychological literature, where self‐harm is demarcated within the ‘biopsychosocial' model [5]. Thus, self‐harm is seen to address emotional dysregulation, an intrapersonal function, alongside interpersonal motivations, such as communicating distress [7, 8]. Discriminatory interpretations of interpersonal reasons can view self‐harm as an attempt to ‘control’ situations, or ‘manipulate’ others [7, 9, 10]. Sociocultural factors and/or personal characteristics, such as being a cisgender woman, LGBTQIA+, and having experienced sexual abuse and/or other trauma, are seen as contributing factors [7]. This multi‐dimensional understanding seeks to capture social and psychological complexity [3].

However, in situating itself as objectively, and morally, able to define what mental ‘illness’ is, and contain the people labelled thus, psychiatry retains [11] social and political power [12, 13]. Therefore, psychiatric biomedical conceptualisations permeate and dominate mental health knowledge and clinical practice [12, 13, 14]. Therefore, whilst a biopsychosocial approach is thought to encapsulate social experiences sufficiently, instead they remain ‘*relegated to the role of triggers or exacerbators of a [genetic] vulnerability*’ [15]. Similarly, emotional antecedents to self‐harm are constructed as discrete categories belonging to an individual [16], overlooking social intentionality [17, 18] and subjective heterogeneity [16, 19]. This, combined with how the biopsychosocial separates human experience into distinct boxes, negates complexity [20] and individual ‘abnormality’ remains centralised [21]. Thus, in contemporary understanding, sociocultural context is not denied but rather treated, as the sociologist Steggals notes, ‘*as a façade, and only as long as we don't ever use it as an actual explanation for some pattern of life*’ [1].

Conversely, since the 1980s, survivors of psychiatry [12, 22] emphasise going beyond ‘symptoms’ [12], ‘*put[ing] the colour into what otherwise might be monochrome portrayals of human distress and resilience’* [23]. Likewise, those with a more critical perspective of lived experience (LE) [1] of self‐harm assert that engagement is not only *informed* by social circumstances but inseparable from it [24], and mediated by ongoing experiences of inequity, such as trauma, abuse, relational and systemic violence, both outside and inside of psychiatry [12, 21, 25, 26, 27]. In this light, self‐harm ‘*manage[s] the distress this world generates*’ [27] as ‘*a sane response when people are gagged in order to maintain the social order’* [22]. This is supported by self‐harm sociological literature that explores how self‐experience begins with, as the scholar Ahmed asserts, the ‘*outside in’* rather than the ‘*inside out*’ [19, 26, 28, 29, 30].

### 1.1 Rationale for the Present Study

We acknowledge that there is a plurality of perspectives about self‐harm [5, 31]. However, as Chandler (2016) suggests, ‘*the ‘reality’ of self‐harm is very much contested*’, and so, it can be difficult to conceptualise self‐harm [37]. In comparing survivor perspectives to psy‐knowledge, we uncover what Donnelly (2024) discusses as an ‘*epistemic discrepancy*’ [32]. This tension becomes clearer when considering that when in contact with mental health services, many feel that ‘*psychiatry has only ever been interested in my ‘behaviour’*’ [9], leading to people with LE feeling harmed, frustrated, invalidated and misunderstood [21, 22, 24, 27]. This highlights the need for further research to centralise LE, especially for views that are underrepresented [12, 21, 26]. However, because they critique the psychiatric hegemony, these perspectives may be delegitimised and excluded from contributing to mainstream understanding [12, 32, 33, 34, 35, 36], working as epistemic injustice [13, 22, 24, 27, 37, 38, 39, 40].

These issues extend to Patient and Public Involvement (PPI), which can be inauthentic and tokenistic, potentially harming those involved [41, 42, 43, 44]. Examples of inauthentic inclusion may involve being ‘invited’ into research spaces without having meaningful control over research, co‐optation of emancipatory initiatives, such as co‐production [42, 43], and researchers being resistant to power share and welcome viewpoints that challenge one's own [41, 44, 45]. Conversely, authentic inclusion of LE may commit to social justice, including developing alternatives to psy‐knowledge and treatment [21, 46], and researchers, including those with LE, being reflective, transparent and accountable for epistemic privilege [44, 46, 47]. Consequently, we sought to transparently apply a survivor approach to this research, where people with LE had control over the research lifecycle to prioritise solidarity and, hopefully, broaden understanding to enact social change [12]. A social justice approach, survivor‐controlled research has its foundations in critical theory [48], valuing first‐person perspectives [14, 49] and typically prioritises trauma‐ and social‐based understandings [26].

### 1.2 Research Objectives and Aims

This integrative review (IR) systematically explores, analyses and interprets self‐harm research through LE perspectives, within a survivor approach and employs participatory methodology. Other reviews of self‐harm have not utilised this approach [5, 50, 51] and so, to the authors' knowledge, it is the first of its kind. IRs differ from conventional systematic methods by seeking to enhance theories to broaden understanding [52]. The following review question, ‘*How is self‐harm conceptualised?’*, and objectives, as developed with PPI peers (SB, GJ and EM), were employed:

Research objective (RO)1: To explore self‐harm construction across the included literature, considering how researchers across different epistemological standpoints explored self‐harm.

RO2: To critically consider whether and how the construction and perception of self‐harm in clinical and academic research and practice may impact self‐concept, ‐perception and understanding in people with LE.

RO3: To critically explore the LE of people who self‐harm.

RO4: To deconstruct the trajectory of self‐harm.

## 2 Methods

### 2.1 Summary

IRs promote theory building by systematically collating evidence with different research designs to broaden conclusions [52, 53, 54]. This allows for alternative research methods, e.g. autoethnography, to be synthesised, directly accessing multiple perspectives to increase inclusivity [55]. To maximise transparency and replicability, the protocol was registered on PROSPERO (CRD42024531540) in April 24, and the IR was guided by best practice guidance and reporting qualitative research [52, 53, 54]. The IR followed five stages [55], as shown in Table 1.

**Table 1:** Summary of integrative literature review methodology [55].

Steps of the IR	Details of step
Step 1: Composing a review question	A review question, guided by the SPIDER framework, was developed to outline inclusion/exclusion criteria and decide upon structure of the search strategy, including keywords.
Step 2: Sampling the literature	The search strategy was applied to five electronic databases, MEDLINE, EMBASE, PsycINFO, CINHAL and Global Health databases in Apr‐26. All titles and abstracts were screened and 36 full text documents addressing review questions and objectives were selected. Rayyan, an online screening tool, was used to review and select literature according to inclusion criteria.
Step 3: Critical appraisal	A thorough and comprehensive evaluation of the transparency, rigour and inclusivity of each relevant study was conducted using a combination of adapted Equity, Diversity and Inclusivity (EDI) framework, adapted Critical Appraisal Skills Program (CASP) for qualitative research (2024) and Guidance for Reporting Involvement of Patients and the Public – Short Form (GRIPP2‐SF) [56].
Step 4: Data Extraction and Synthesis	Data relating to the article title, authors, research objectives, main findings, data analytical process and theoretical approach, if stated, and methodology were extracted. Each study was transferred to NVivo to be analysed using reflexive thematic analysis, situated with a survivor approach and social constructionism. Entire articles were analysed: how researchers constructed self‐harm in the introduction, discussion, conclusions and how quotations from LE were described and understood addressed RO1 and RO2. Narrative data were analysed to address RO3 and RO4. Two themes were produced: Theme 1, *What is self‐harm*? addresses RO1 and RO2, and Theme 2, *The web of self‐harm*, addresses RO3 and RO4.
Step 5: Presentation of Findings and Discussion	Entire integrative review was documented, draft written by CdCL, editing by all co‐authors. Our, CdCL, SB, GJ and EM, collaborative interpretations as drawn from our LE is explored as *The Liminality of Self‐harm*.

### 2.2 Theoretical Approach

We adhere to a survivor approach. However, we assert there is not a ‘correct’ survivor approach, rather, all (LE) researchers should be flexible and adaptable to the possibility of alternatives [57]. Therefore, we present an interpretation, drawn from UK survivor literature and Canadian Mad Studies [12, 14, 25, 46, 49]:

- Led by people with LE of the topic under study [58, 59], where first‐person perspectives are valued as equitable to psychiatric understanding, offering an opportunity for what may be deemed closer, more authentic insight [49]. A survivor approach typically prioritises social‐ and trauma‐based understandings, in line with a trauma‐informed approach (TIA) [60, 61].
- Seeks to move towards collective understandings and dismantling the power differential between researcher and ‘researched’ [25]. Therefore, collaborating with others with LE through participatory methodology is best practice to enhance inclusivity and multivocality [25]. To enhance this, all researchers, including those with LE, should be ‘*reflexive and self‐critical*’ [46], considering how mental distress is shaped by how we are situated in sociocultural and political life, and we, as researchers, should work towards being accountable to and aware of one's epistemic privilege [45, 58, 62]. Thus, ‘*the way in which identity is used*’ [26] is crucial.

Our epistemology was social constructionism, where subjective experience is seen as occurring (with)in and developing across social, historical and relational discourses and interactions [21, 63, 64]. This is aligned with a survivor approach as, as suggested by Sweeney, ‘*[LE] researchers should work towards developing a social constructionist survivor standpoint, which challenges dominant [psy] discourses*’ [5]. Ultimately, we are seeking to reconnect ‘*people's distress to their experiences and circumstances*’ [65], working towards empowerment where people with LE feel they are recognised within the knowledge produced and their voices are heard [21].

### 2.3 What Did the People With LE Control in the IR?

Here, we explore the application of a survivor approach, guided by Faulkner's *Ethics of Survivor Research* [46] and the Survivors' Voices *Charter for Engaging Abuse and Trauma Survivors* [47]. As this forms part of CdCL's doctorate, we focus on how they worked with PPI peers. CdCL has LE of self‐harm through eating distress and self‐cutting [24, 66, 67], complex trauma, mental health service use and has worked as a general nurse [24]. SB, GJ and EM were PPI members on the Lived Experience Advisory Panel (LEAP) attached to CdCL's doctorate.

#### 2.3.1 Reflexivity

Reflexivity involves continuous, critical introspection of one's positionality to support knowledge development [68], increasing methodological transparency, integrity and rigour [69, 70, 71]. This section presents an overview of CdCL's early and developing understanding of self‐harm.

CdCL's interest in investigating self‐harm comes from lived and professional experiences. For example, in feeling minimised and unheard by others, and seeing people under mental health services facing similar situations, CdCL's aim is to work towards to developing knowledge with others with LE, so that, hopefully, they feel recognised in the work produced [72]. Upon beginning the doctorate, CdCL engaged extensively with self‐harm literature [27, 73, 74, 75, 76, 77] and with colleagues with LE and across disciplines. Through a reflexivity diary (from Oct‐23), they reflected on their self‐harm as a ‘thing’ that becomes ‘mine’ [1, 78] that, for them, feels imbued with social meaning and relational distress [24]. Thus, they do not consider it as solely an individualised behaviour, even though this is prioritised in clinical practice [4, 79]. These reflections guided the first doctoral study [24], as well as the initial RO for this review. Further reflective work by CdCL is also available elsewhere [24, 45, 80, 81].

CdCL's understanding has developed through the analytical process and by reflecting collaboratively with doctoral supervisors (ML, UF and AS), tertiary supervisor (PS), a sociologist who researches self‐harm, external clinical supervisor (KM), who works as a DBT/EMDR psychotherapist, and LEAP members (SB, GJ and EM). CdCL reflected that they previously adhered to clinical language to understand themselves, and others, such as intellectualising emotionality as ‘dysregulated’ [16, 82]. In doing so, CdCL found they could not connect meaningfully to how social inequality and harm moulds self‐experience, leading to a dispassionate, judgemental stance on their, and others' distress [24, 80, 81]. However, CdCL now identifies their (self‐)experiences – including intense, vivid and amorphous emotionality ‐ as *given to* them through the social world [83].

#### 2.3.2 Patient and Public Involvement Through the Lived Experience Advisory Panel (LEAP)

In early discussions with doctoral supervisors (AS and ML), it was concluded that it would be unethical to rely solely upon CdCL's LE [84]. Therefore, participatory methodology was employed to enhance inclusivity, mitigate proprietorial ownership and testimonial injustice [85]. PPI was implemented early (in Feb‐24) to ensure co‐authors with LE could influence research direction. Underserved groups (particularly those belonging to racialised communities) were prioritised in recruitment to ensure a diverse panel. CdCL collaborated with SB, GJ and EM – who were paid as per National Institute of Health Research (NIHR) guidance [86] – in meetings occurring online every 3–4 months.

#### 2.3.3 Overview of LEAP Meetings

In early meetings, LEAP members explored self‐harm as coming ‘*from somewhere/something that we picked up along the way in our lives’*, but that ‘*self‐harm is looked at in a certain way in a certain demographic’* where ‘*people get put into a box and not taken seriously’*. In being involved in PPI, LEAP members wanted to help others with LE, learn about different perspectives, make research more relevant and have their voices heard. LEAP members suggested regular, clear communication, a gentle approach to sharing personal experiences and being respected as a colleague, to combat tokenism.

CdCL's approach to collaboration with peers was guided by survivor [46, 47] and TIA PPI frameworks [84, 85, 87]. Thus, they aimed for a strengths‐based, peer‐to‐peer approach towards mental distress [47]. In practice, this means CdCL views self‐harm not as a ‘clinical risk’ but a mark of resilience [27, 47]. They also try to dialogically reflect upon, and share, LE to be transparent and critically develop their knowledge, working towards multivocality [80].

CdCL initially suggested that they develop a LE‐derived definition from the literature. However, EM argued that this would risk excluding marginalised people by further compartmentalising the phenomenon. CdCL reflected that whilst this initial RO felt relevant to them – as assigned female at birth, white, middle‐class, and a researcher who is already represented, taken seriously and able to navigate systems of power because of these social identities ‐ it would have risked ‘*speaking for’* others, categorising LE excessively [87] and thus contributing to a clinical, colonial lens [62]. In response, the LEAP collaborated to introduce RO2.

Overall, this partnership working evolved over time, reflecting a holistic approach. Our experience of PPI is that it often involves tokenism and so, we sought to overcome this [88] by collaborating to create a space of solidarity and reciprocity. For peer co‐authors, this meant ownership over their words and freedom over the project, prioritising their LE and thus, offering the opportunity for unheard voices to equally influence data interpretation, discussion and drafts [85, 89].

### 2.4 Search and Eligibility Criteria

The Sample, Phenomenon of Interest, Design, Evaluation and Research Type (SPIDER) framework for qualitative evidence synthesis was used to develop eligibility criteria [90] (Table 2).

**Table 2:** Finalised eligibility criteria with associated search terms.

SPIDER	Eligibility criteria	Search terms
Sample	Participants were people with lived experience of self‐harm, of any age. They could be diagnosed with any psychiatric condition or be from a non‐clinical population.	‘Young adult*’ OR ‘adolescent*’ OR ‘Service‐user’ OR ‘Consumer*’ OR ‘expert‐by‐experience" OR ‘older adult*’
Phenomenon of Interest	Self‐harm defined as ‘*as any act that causes direct harm to the body but (.) where the focus and purpose of the act is this harm itself and not some other goal such as decorative body modification or suicide*’. [91]	‘Self‐harm’ OR ‘non‐suicidal self‐injur*’ OR ‘self‐injur*’ OR ‘self‐injur* behaviour*’ OR ‘Parasuicide’ OR ‘self‐mutila*’ OR ‘NSSI’ OR ‘DSH’ OR ‘deliberate self‐harm"
Design	Included articles were peer‐reviewed, qualitative studies exploring self‐harm. Research was included if using open‐ended questions, focus groups and interviews were used. Exclusions included the use of closed questionnaires, message boards and/or structured interviews. This is because these methods could limit the space with which people with LE could discuss what feels important and relevant to them. Research must have implemented PPI, as per the NIHR INVOLVE Levels of Involvement. This was to maximise the inclusion of LE in the research lifecycle. Where PPI was not explicitly discussed in literature, corresponding authors were emailed to clarify. Conversations with colleagues with LE or pilot interviews with people with LE, were not considered PPI and thus, excluded.	‘Qualitative*’ OR ‘interview*’ OR ‘thematic*’ OR ‘ethnograph*’ OR ‘grounded theory’ OR ‘phenomenolog*’ OR experiential OR ‘focus group*’ OR ‘discourse*’ OR ‘conversation analysis’ OR ‘framework analysis’ OR ‘narrative’ OR ‘account*’ OR hermeneutic OR ‘Mixed method*’ OR ‘mixed‐method’ OR ‘semi‐structure’ OR ‘semi structure’ OR ‘IPA’ or ‘Interpretative*’ OR ‘content analysis’ OR ‘guided discussion’ OR ‘group discussion’ OR open‐ended OR ‘open‐ended’
Evaluation	Evidence must examine self‐harm experiences and/or conceptualisations, as led by the first‐person perspective. Evidence was excluded if it discussed recovery, e.g. not on the meaning and lived reality of self‐harm. Further, evidence was also excluded if it only examined clinicians’ or carers’ experiences only, focused on one selected aspect of self‐harm and/or assessed quality of care and treatment. Overall, evidence must have created space with which people with LE could discuss key areas of importance to the experience of self‐harm, such as its meaning, subjectivity and function.	‘Concept* OR ‘defin*’ OR ‘understand*’ OR ‘mean*’ OR ‘Lived experience’ OR ‘first‐hand’
Research Type	Research could be empirical or non‐empirical qualitative research. They must have implemented critical analysis. Evidence was included if it was published in English between Jan‐2000 to date.	Not applicable

**Figure 1:**
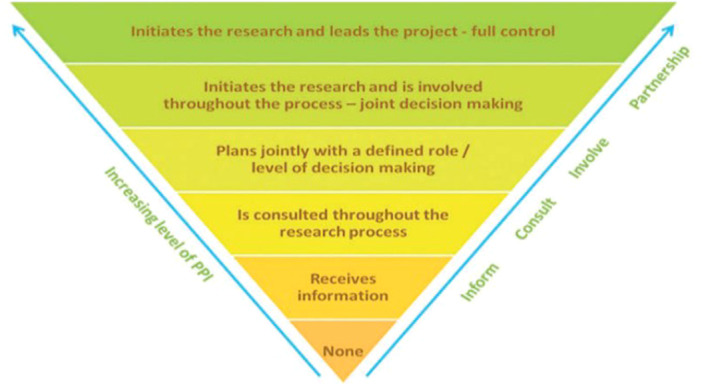
NIHR INVOLVE – levels of involvement [92].

Studies must have implemented Patient and Public Involvement (PPI). PPI can be defined as research ‘*carried out with or by members of the public rather than to, about, or for them*’ [56]. To determine PPI, we followed the NIHR ‘Levels of Involvement [92]. This describes how PPI can be carried out: being LE‐led, collaborating with LE co‐authors and/or working alongside members of a project advisory group [84, 92, 93] (Figure 1).

We acknowledge that some researchers have undisclosed LE, and we do not wish to invalidate or suggest that disclosure is necessary to ensure research validity. Instead, we were interested in exploring how self‐harm was discussed by researchers situated across epistemological standpoints, including those who critically explored LE. Self‐harm terminology is extensive. All terms were included to synthesise multiple perspectives, but we define self‐harm as ‘*direct harm to the body, but (…) where the focus and purpose of the act is this harm itself*’ [94], to avoid ‘stripping the motivation’.

### 2.5 Searches and Selection

**Figure 2:**
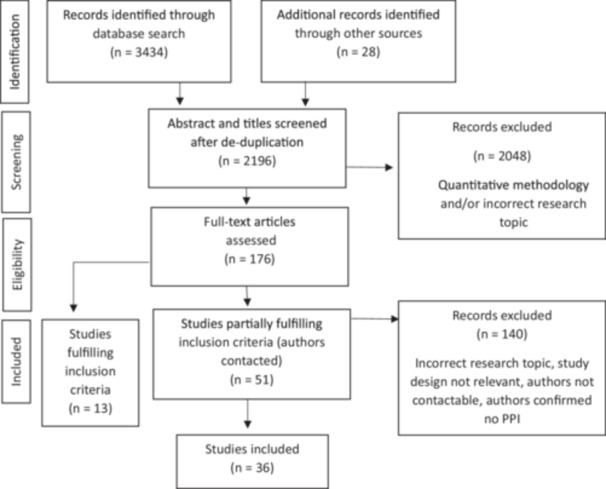
PRISMA diagram.

Screening and article selection was completed by CdCL. A search using MEDLINE, EMBASE, PsycINFO, CINAHL and Global Health databases was conducted in Apr‐24, then repeated in Mar‐26, yielding 2196 articles, after de‐duplication. Additional searching through forward/backward citation and grey literature search engines, such as GreyCat, was conducted, yielding 28 additional articles. Titles/abstracts were screened by CdCL, and 148 records were selected for full‐text screening. 21 articles fulfilled inclusion criteria. 50 articles required additional information from author(s) about whether PPI was used. 36 articles were included (Figure 2).

### 2.6 Data Extraction

Study characteristics, such as aim(s), country of origin, study design, data collection method, data analysis method, recruitment, sampling method, PPI method, sociodemographic information and theoretical approaches were extracted (see Table 3).

**Table 3:** Data extraction of included studies.

No	Author(s)	Origin	Aim(s)	Participants	Sampling	Methodology	Discipline, data analysis and theoretical approach	PPI	CASP
1	Boyce (2021)	UK	To examine the lived experiences of self‐harm in adults 25 to 60 years.	18 participants (15 = cisgender women, 2 = cisgender men, 1 = fluid male), aged 26 – 58 years. 8 = heterosexual, 4 = bisexual, 2 = asexual, 2 = gay, 1 = lesbian and 1 = demi‐sexual.	Purposive sampling	Open‐ended online questionnaires enabling participants to share their individual experiences of self‐harm, including its role, changing experience and any access to support.	Social science, thematic approach, no underlying epistemological approach given.	Consultation with mental health advisory panel on study design, PIS and survey questions	16
2	Brown et al. (2022)	UK	To explore the psychological processes that are associated with shame in those who engage in NSSI, focusing on how feelings were triggered, how they were processed and responded to.	6 participants (5 = cisgender women, 1 = non‐binary), 17 to 23 years old.3 = White British, 1 = Other Mixed Background and 2 Asian British Pakistani. 5 = heterosexual, 1 = asexual.	Purposive sampling	Mixed method study, combining experience sampling methodology (ESM) diaries with semi‐structured interviews that were used to elaborate on instances from the diaries that identified feelings of shame.	Psychology, thematic analysis, critical realist epistemological stance.	LE collaborator as co‐authors and consultation with advisory panel in study design and focus	17
3	Chandler & Simopoulou (2021)	UK	To interrogate the construction of gendered meanings of self‐harm within collaborative arts‐based workshops	12 participants (5 = cisgender men, 7 = cisgender women), aged 21‐37 years.	Purposive sampling	Qualitative workshops (90 min), group discussions and art making, in response to surveys and qualitative interviews that may ‘fix meaning’, resulting in ‘standardised explanations’ for self‐harm.	Sociology, feminist post‐structural qualitative inquiry.	Led by person with LE	18
4	Chandler (2012)	UK	To explore self‐harm as an embodied emotional practice	12 participants (5 = cisgender men, 7 = cisgender women), aged 21‐37 years.	Purposive sampling	Qualitative interviews – two per participants – using a ‘life‐grid’ and then encouraging participants to bring their own themes to the second interview, with a collaborative approach.	Sociology, thematic and narrative analysis, sociological approach	Led by person with LE	16
5	Chandler (2013)	UK	To explore the nature of physical versus emotional pain in self‐injury	12 participants (5 = cisgender men, 7 = cisgender women), aged 21‐37 years, 10 = white, 2 = unspecified.	Purposive and snowball sampling	Qualitative interviews – two per participants ‐ on two occasions using a ‘life grid’ to structure discussions. The second interview was initially supported by written summaries from the first, aimed to have a collaborative approach.	Sociology, narrative analysis, combining a narrative approach with phenomenology	Led by person with LE	17
6	Chandler (2014)	UK	To apply Frank's typology of illness narratives to self‐injury, focus on bodily aftermath of self‐injury: scars, wounds and marks.	3 participants (2 = cisgender women, 1 = non‐binary), aged 21‐26 years. All white.	Purposive sampling	Qualitative interviews, two interviews per participants, with life‐story approach in first and then discussing self‐harm more explicitly in the second.	Sociology, narrative analysis/illness narrative, sociological theorisation	Led by person with LE	17
7	Donskoy & Stevens (2013)	UK	To explore people's first episode self‐wounding.	8 participants, 6 = cisgender women, 2 = cisgender men; aged between 21‐65. All White British.	Purposive sampling	Qualitative study, semi‐structured interviews, exploring the first episode of self‐harm.	Social science, thematic analysis; illness narrative (Frank, 1995), survivor approach	Survivor‐led	17
8	Edmondson et al (2018)	UK	To explore the acceptability of using photo‐elicitation and to discuss reasons why adults self‐harm	11 participants (5 = cisgender women, 6 = cisgender men), aged 19‐50 years.	Purposive sampling	Qualitative interviews (40‐120 min), using photo elicitation.	Psychology, polytextual thematic analysis, research paradigm not stated	Consultation in research design	16
9	Gosling et al. (2023)	UK	To understand the self‐harm experiences of transgender young adults	11 participants, aged 18 to 30 years, all identified as non‐binary, 10 = White; 1 = Arabic.	Purposive sampling	Qualitative study using interviews (60 min), with participants.	Clinical psychology, grounded theory, constructivist epistemology	Consultation in research design	19
10	Gunnarsson (2021)	Sweden	To critically illustrate the interplay between shame and self‐harm	The author only, who has a LE of self‐harm	Not applicable	Critical reflective essay, or ‘first person account’ of self‐harm and shame using personal diaries.	Sociology	Led by a person with LE	N/A
11	Gunnarsson (2021)	Sweden	To explore shame and self‐harm	The author only, who has a LE of self‐harm	Not applicable	Critical reflective essay using personal diaries of shame and self‐harm.	Sociology	Led by a person with LE	N/A
12	Gunnarsson (2022)	Sweden	To explore the meaning of self‐harm and self‐harm scars related to cutting	The author only, who has a LE of self‐harm	Not applicable	Critical reflective essay considering the meaning of self‐harm scars.	Sociology	Led by a person with LE	N/A
13	Gunnarsson (2023)	Sweden	To explore the LE of self‐injury and understand interplay of age, gender and psychopathology	The author, with LE, and four people (4 = cisgender women, 1 = non‐binary), aged 35‐51 years with LE, all were white.	Purposive sampling	Autoethnography with interviews with five women with LE, including the author.	Sociology, deductive thematic analysis, autoethnographic approach	Led by a person with LE	18
14	Gunnarsson (2024)	Sweden	To explore the relationship between self‐destructiveness and identity	The author only, who has a LE of self‐harm	Not applicable	Critical reflective essay considering the meaning of images and thoughts of self‐destruction	Sociology	Led by a person with LE	N/A
15	Gurung (2018)	Australia	To explore self‐harm as embodied healing, survival and self‐creation	The author only, who has a LE of self‐harm	Not applicable	Critical reflective essay considering self‐harm as embodied existential angst.	Sociology	Led by a person with LE	N/A
16	Heney (2020)	UK	To explore agency as related to self‐harm by discussing within feminist frameworks	The author only, who has LE of self‐harm	Not applicable	Critical reflective essay exploring feminist concepts of agency and embodiment in relation to self‐harm practice	Social sciences, sociology	Led by a person with LE	N/A
17	Jackman et al. (2018)	USA	To explore the self‐harm experiences of transmasculine spectrum individuals.	18 participants, 17‐38 years, all identifying as transmasculine (transmen = 10; non‐binary = 8), 7 = non‐Hispanic white, 2 = African American, 6 = Hispanic, 3 = Other.	Convenience sampling	Qualitative study using semi‐structured interviews lasting 33‐92 min.	Psychiatric nursing, directed content analysis, Nock's theoretical model of self‐harm, and minority stress model	Consultation with advisory panel on all aspects of the study, not all members had self‐harmed	16
18	Lockwood et al. (2021)	UK	To explore the salience and context of multi‐dimensional impulsivity, emotional and cognitive influencers on self‐harm.	15 participants (1 = men, 14 = women), aged between 16 to 22, 10 were white and 5 were described as ‘mixed ethnic heritage’.	Convenience sampling	Qualitative study using interviews, lasting between 45‐56 min, and a card‐sorting task.	Psychology, thematic analysis, small ‘q’ approach to TA, no epistemological position or paradigm stated	Consultation with advisory panel on design and methods	14
19	Lundwall et al. (2025)	USA	To explore the perspectives of autistic women who self‐injure	23 participants, (all cisgender women), aged 18 to 41 years, 22 = white, 1 = Asian (no specification).	Purposive sampling	Mixed methods with qualitative component using semi‐structured interviews focusing on NSSI and suicidal thoughts and behaviours.	Psychology, collective case study approach, no epistemological approach specified.	Consultation with two autistic women with LE of self‐harm on data analysis	19
20	Miller et al. (2021)	UK	To investigate how adolescent girls make sense of self‐harm.	9 participants (all cisgender women), aged 13‐17, current engagement.	Purposive sampling	Qualitative study using semi‐structured interviews lasting approximately 60 min.	Psychology, interpretative phenomenological analysis (IPA), phenomenology	Consultation with advisory panel on design, questions and protocol	17
21	Morris et al. (2015)	UK	To explore emotion for those with self‐harm and have complex emotional difficulties	8 participants (1 = men, 7 = cisgender women), aged 21 to 51 years, all White British.	Purposive sampling	Qualitative study using semi‐structured interviews (40‐120 min).	Clinical psychology, narrative analysis, no epistemological position stated	Consultation with advisory panel in all stages	17
22	Mughal et al. (2023)	UK	To explore the functions of self‐harm in young people	13 participants, (12 = cisgender women, 1 = transgender man), aged 19 ‐25 years. 7 = White British, 3 = Mixed Ethnicity, 1 = White American, = 1 Asian British, 1 = unspecified.	Purposive sampling	Qualitative, semi‐structured interviews.	Psychology and medicine, RTA, no epistemological position stated	Consultation with advisory panel on research materials	17
23	Naz et al. (2021)	UK/Pakistan	To explore the LE of adolescents with self‐harm and their reviews on suicide and self‐harm prevention	16 participants (9 = cisgender women; 7 = men), 14‐17 years.	Purposive sampling	Qualitative, semi‐structured interviews.	Psychology and psychiatry, framework analysis, no epistemological position or paradigm stated	Consultation with advisory panel on study design	16
24	Norman et al. (2022)	UK	To explore self‐harm as ‘bad’	8 participants (6 = cisgender women, 2 = other). 3 = Black British, 1 = Asian, and 2 = mixed ethnicity, 2 = white, 2 = unspecified, aged 18‐29.	Convenience sampling	Qualitative, semi‐structured interviews, with optional photo‐elicitation.	Psychology, IPA	Consultation in design with person with LE	17
25	Norman et al. (2023)	UK	To explore functions of self‐harm in young adults with alexithymia	As above with Norman et al. (2022).	Convenience sampling	Qualitative study using semi‐structured interviews (49‐100 min), with optional photo‐elicitation,	Psychology, IPA	Consultation in design with person with LE	17
26	Pollock et al. (2021)	Ireland	To explore self‐harm onset, repetition and cessation	9 participants (5 = cisgender women, 3 = cisgender men, 1 = did not identify with gender) aged 16‐18 years.	Purposive sampling	Qualitative study using semi‐structured interviews (25‐60 min).	Social work, thematic analysis, no epistemological position or paradigm stated	Consultation in design with young person with LE	16
27	Rebbettes & Bacon (2025)	UK	To explore the psychosocial triggers and experiences contributing to self‐harm, the thoughts and feelings surrounding it and the broader impact of self‐harm on the lives of autistic women.	7 participants (all cisgender women), aged 21 to 40 years. No other information given.	Purposive sampling	Semi‐structured interviews lasting between 36‐74 min and sought to explore external and interoceptive influences of self‐harm, drawing insight from existing research and writings by autistic women.	Psychology, reflexive thematic analysis, neurodiversity paradigm, including a strengths‐based approach to autism	Autistic‐led research	17
28	Rosendot & Lewis (2020)	Canada	To gain understanding of the experience of NSSI disclosure	17 participants (16 = cisgender woman, 1 = man), aged 18 to 22 years.	Convenience and purposive sampling	Semi‐structured interviews lasting 30 to 50 min, exploring participants’ experiences of disclosing or not disclosing NSSI.	Psychology, thematic analysis, no underlying epistemology explored	Co‐led with person with LE	16
29	Russell et al. (2010)	UK	To explore men's self‐harm	4 participants (all men), White British, aged between 37‐58 years.	Opportunistic sampling	Qualitative study using semi‐structured interviews twice (90 min).	Psychology, Hermeneutic phenomenology	Consultation with advisory panel on design	19
30	Sabo et al. (2025)	Australia	To explore experiences of self‐harm and mental health care received for self‐harm or suicidal ideation among autistic youth.	7 participants, (3 = cisgender women, 2 non‐binary, 1 = transsexual, self‐identified, 1 as transwoman), aged 15 to 23 years. No other information given.	Purposive sampling	Semi‐structured interviews lasting between 30 – 90 min, exploring experiences of self‐harm, amongst other topics.	Reflexive thematic analysis, constructivist position	Led by autistic woman	15
31	Simopoulou & Chandler (2020)	UK	To explore self‐harm as meaningful self‐care through the body	88 participants aged between 13‐26 years.	Not applicable	Critical reflective essay exploring accounts memoirs and personal accounts of self‐harm.	Not applicable	Led by person with LE	N/A
32	Stänicke (2021)	Norway	To explore self‐harm among adolescents	19 participants, (all cisgender women), aged 13‐18 years.	Purposive sampling	Qualitative study using semi‐structured interviews with adolescents.	IPA	Consultation with advisory panel in design and data analysis	16
33	Troya et al. (2019)	UK	To explore how older adults experience self‐harm	9 participants (6 = cisgender women, 3 = men), aged 60 – 72 years and 4 healthcare workers with LE (3 = women, 1 = man),	Purposive sampling	Qualitative study, semi‐structured interviews, with 8 follow‐up interviews.	Thematic analysis, no epistemological position or paradigm stated	Consultation with advisory panel for design and data analysis	18
34	Williams et al. (2023)	UK	To explore the views of young LGBTQ+ people's self‐injurious behaviours.	19 participants, (11 = cisgender, of which 1 = man, 10 = women, 6 transgender (4 transmen; 2 transwomen), and 2 were non‐binary) aged 16 to 25. No ethnicity information given, majority identified as from UK.	No information given	Cross‐sectional qualitative study using semi‐structured interviews, lasting between 45 to 89 min, exploring self‐harm ideation and behaviour.	Thematic analysis	Led by a person with LE and consultation on topic guide with an advisory panel with people with LE	19
35	Witcher et al. (2025)	UK	To explore what maintains repetitive engagement in self‐harm, whether repetitive self‐harm is experienced as an addictive behaviour, and if so, if it is maintained by similar processes to other addictive behaviours.	15 participants (2 = cisgender men, 13 = cisgender women. Ashkenazi (Jewish) = 1, Black or Black British – African = 1, 1 = Malay, White = 12, aged 20 to 61 years old.	Purposive sampling	Semi‐structured interviews supported by audio vignettes drawn from focus group with people with LE of addiction, where responses were edited into short audio vignettes. These were presented in a pilot interview with a person with LE to determine acceptability.	Clinical psychology, constructivist grounded theory, critical realist epistemological stance	LE‐led with consultation with two expert‐by‐experience advisors throughout the project, including on recruitment, data collection, analysis and impact	18
36	Woodley et al. (2021)	UK	To understand how people with LE conceptualise risk	10 participants (3 = men, 7 cisgender women), aged 19‐45 years.	Convenience sampling	Qualitative study using semi‐structured interviews (28‐129 min).	Psychology, IPA	Co‐authorship with a person with LE	18

### 2.7 Quality Assessment

No articles were excluded based on quality. Quality assessment was conducted by CdCL using Critical Appraisal Skills Program (CASP) for qualitative research (2024) [31], (Supporting Material (SM)1; Guidance for Reporting Involvement of Patients and the Public – Short Form, GRIPP2‐SF) [56] (SM2); and an adapted Equity, Diversity and Inclusion (EDI) framework guided by EDI principles and values [47, 95, 96] (SM3).

### 2.8 Data Analysis

Reflexive thematic analysis (RTA) followed six stages, and aligned to a ‘Big Q’ approach, with a deductive, latent orientation [97]. RTA was chosen because it explicitly centralises researcher subjectivity, complementing our approach [97, 98]. The ‘Big Q’ approach views knowledge as ‘*partial and situated’* and *‘subjectivity as a resource for research*’ [99]. Therefore, coding and theme development was determined by perceived salience [97, 99] and contextualised within the LE of CdCL, SB, GJ and EM and complemented by CdCL's nursing experience [100]. Stages 1 to 3 involved CdCL closely examining literature to identify preliminary patterns, with line‐by‐line coding and then initial theme development. Narrative data, such as critical commentaries written by researchers with LE and quotations from people with LE, was analysed to address RO2, RO3 and RO4. The ways in which researchers constructed self‐harm, for example, how researchers explored participant quotations, and introduction/discussion sections, addressed RO1 and RO2 (see SM4 and accompanying methodological paper, in peer review [80]). Early themes were presented to peer co‐authors for interpretation and refinement (Stages 4 and 5) (SM5 and SM6). Stage 6 involved report generation by CdCL with editing by co‐authors.

## 3 Results

Thirty‐six articles were eligible (Table 3). Twenty‐six were qualitative studies, using semi‐structured interviews, open‐ended questionnaires and a collaborative workshop. Other studies were critical commentaries.

Most studies referred to ‘self‐harm’. Other terms used were ‘non‐suicidal self‐injury’ (NSSI), ‘self‐injury’, ‘deliberate’ or ‘repetitive self‐harm’ and ‘self‐wounding’. Methods included self‐cutting, restrictive and binge‐eating, substance use, self‐bruising, ‐punching, ‐poisoning, ‐burning, interfering with wound healing and hair pulling (SM7). Participants identified as cisgender women (*n* = 251, 70%), cisgender men (*n* = 51, 14%), non‐binary people (*n* = 26, 8%), transwomen (*n* = 3, 1%), transmen (*n* = 17, 5%), and other (*n* = 5, 2%). Participant ages ranged from 14 to 72 years. Most studies recruited from adolescence to middle adulthood, others recruited from adolescence to young adulthood, only from adolescence, young to middle adulthood, young adulthood only, across the lifespan and older adults only.

Twenty‐three were from the UK, five from Sweden, one from Norway, three from Australia, two from the US, one from Ireland, one from Canada and one jointly between the UK and Pakistan. Only fifteen studies reported on sociodemographic information relating to race and ethnicity. Of these, participants were mostly white British, European or American (*n* = 122, 83%), with only eight studies including people from racialised communities [101]. Of these, people identified as Mixed Ethnicity (*n* = 8, 0.8%), unspecified or undisclosed (*n* = 5, 3.8%), Arabic = (*n* = 1, 0.8%), African American (*n* = 2, 1.6%), Black British (*n* = 4, 2.5%), Asian, no specification (*n* = 2, 1.6%), Asian British (*n* = 1, 0.8%), Ashkenazi (Jewish) (*n* = 1, 0.8%), Malay (n = 1, 0.8%), Other Mixed (*n* = 1, 0.8%).

### 3.1 Overview of Themes

**Figure 3:**
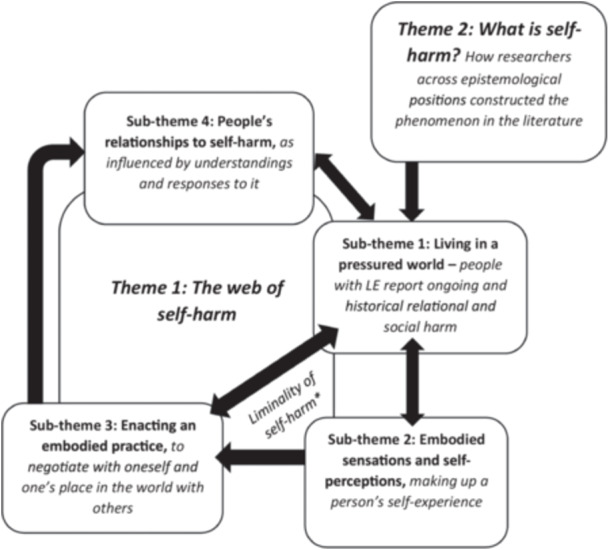
Visual overview of themes (main themes in ***bold italics***; sub‐themes in **bold**).*Please see Section Conclusion, 3 **: Discussion** *for exploration of the ‘Liminality of self‐harm’*.

Two themes were developed. **Theme 1: What is self‐harm?** explores how researchers constructed self‐harm when exploring it in the literature, addressing RO1 and RO2 [102]. **Theme 2: The web of self‐harm** addressing RO2, RO3 and RO4, explores self‐harm further, exploring LE data to generate an understanding of a ‘*self‐harm trajectory’* (Figure 3). Codes are indicated by italics.

### 3.2 Theme 1: What Is Self‐Harm? How Researchers Explore the Phenomenon

In this theme, we present and explore self‐harm conceptualisations across the included research, building on work by Chandler et al. (2011) [102].

Self‐harm was typically introduced as a self‐injurious behaviour directed towards one's body [103, 104, 105, 106], excluding suicidal intention [10, 72, 103, 104]. However, many psychological researchers highlighted increased risk for suicide, with some presenting self‐harm as a major public health concern [103, 106, 107, 108, 109, 110, 111].

Self‐harm was framed within certain *identities*, as a ‘*behaviour affecting young people’* [104, 107, 108, 111, 112, 113, 114, 115]. Indeed, most participants were white young women [16, 104, 109, 111, 112, 113, 116, 117, 118, 119, 120, 121], and gendered interpretations were apparent [104, 118, 122]. For example, Miller et al. (2021), a psychological study, explored ‘*socialised differences’* between men and women, comparing ‘internalised/externalised’ distress [104]. Some sociologists, many of whom had LE, explored how gendering self‐harm reinforces it as a ‘*female' act’* [16, 123, 124], minimising so‐called ‘*delicate cutting*’ [123]. Alternatively, it can be argued that ‘*patterns of self‐harm are diverse, sensitive to context, definitions and measures used’*, and Gunnarsson, another sociologist with LE, noted that constructing self‐harm within specific identities renders underserved people, such as older adults and people of colour, ‘*invisible*’ [78].

Researchers, especially those with LE, explored how people who self‐harm are *portrayed negatively* [78, 119, 120, 125, 126]: ‘*manipulative’*, ‘*attention‐seeking’* [113, 119, 123, 127, 128], ‘*lacking intra‐ and interpersonal skills*’ [119], having failed ‘*to develop healthy coping mechanisms to meet’* [127] needs and thought of by clinicians as ‘*risky*’, ‘*maladaptive*’ [127], morally wrong [105, 109, 113, 119, 120, 127]. Likewise, three psychological studies, one of which was LE‐led, acknowledged the stigma surrounding self‐harm [114, 129, 130].

Some of the psychological literature contextualised self‐harm by presenting it as co‐existing with mental ‘illness’ [107, 110, 111, 118, 131], and others situated it as pathological by using the diagnosis ‘NSSI’ [114, 118, 131, 132, 133]. As highlighted by psychological researchers Edmondson et al. (2018), people with LE have critiqued pathologisation as problematising, without seeking to understand [10]. However, many others did not emphasise pathology [10, 103, 116, 117, 119, 120, 123, 124, 125, 127].

Many psychological researchers endorsed ‘*emotional regulation’* as the prevailing function [10, 103, 104, 105, 106, 107, 112, 113, 118, 131, 132], where self‐harm was constructed as ‘*get[ing] relief from negative feelings’* [10, 104, 106, 113, 127, 132, 133]. However, sociologists explored how this ‘*obscures sociocultural contexts’* [16], overlooking how emotionality has social intentionality [16, 116, 120, 125, 127]. Chandler argued that ‘*emotional aspects of self‐harm have been engaged with relatively superficially’* [16]. However, some recent psychological research sought to capture how social life mediates one's sense of self [103, 122, 133, 134]. For example, Brown et al. (2022) explored the social nature of shame [133]. Gunnarsson (2021, 2023), in exploring their LE, expanded upon this, exploring how self‐harm combats shame, upholding relational connectivity but threatens social order [120, 125]. Two other studies, one of which was LE‐led and situated within a strengths‐based approach and the other involving people with LE, sought to explore social distress on autistic women's LE of self‐harm [122, 132], going beyond individual deficiency [78, 117, 119, 120, 125].

Some research noted *inter*personal functions [118, 120], such as connecting, communicating or influencing people [10, 104, 110, 113, 118]. Further discussions of social experiences, such as ‘*trauma’* or difficult childhood experiences, could appear decontextualised [72, 105, 106, 115, 127, 131], such as when only minimally referred to [10, 107, 110], but then implicitly centralising the individual [104, 105, 106, 108, 131], or not referenced at all [111, 112]. For instance, Jackman et al. (2018), underpinned by Nock's inter/intrapersonal model and the Minority Stress Model, noted that an ‘*environment with little support and few resources*’, where self‐harm addressed ‘*stress*’ [131] to contextualise the following:

…I found a thing that helps me and (…) ideally (…) someday there would be a planet where I don't have to do that but (…) like the situation that I'm in now this is the best way for me to survive… [131].

Similarly, Gosling et al. (2023) referred to how invalidating familial environments ‘*underpinned participants’* lack of skill *in processing emotion’* [106] (our emphasis). Likewise, Naz et al. (2021), noted that ‘*self‐harm was closely linked to a situation with which the adolescent* could not *deal*’ (our emphasis), to understand: ‘*My parents were angry with me and were trying to make me tense*’. Ultimately, as recognised by psychological researchers, Norman et al. (2022), ‘*research often suffers from a narrow focus on internal factors’* [113], negating self‐harm's *divergent meanings*, including its ambiguity, as acknowledged by sociologists with LE [78, 117, 119, 127, 128].

### 3.3 Theme 2: The Web of Self‐Harm

‘**The web of self‐harm’**, named by GJ, describes the ‘*self‐harming trajectory*’, exploring how social experiences (**sub‐theme 1**) mould people's self‐experience (**sub‐theme 2**), leading to self‐harm (**sub‐theme 3**), implicating self‐perception, as also influenced by wider social responses (**sub‐theme 4**).

#### 3.3.1 Sub‐Theme 1: Living in a Pressured World

This sub‐theme describes the relationally and socially difficult experiences people with LE are exposed to, exploring the sociocultural context they are situated in.

*Familial and peer harm* was frequently discussed [104, 105, 106, 108, 110, 111, 113, 118, 121, 131], including bullying [10, 73, 104, 106, 107, 110, 115, 122, 134], violence [10, 72, 106, 110, 131], feeling responsible for others or for discord [104, 114, 115, 132], social isolation and rejection [106, 113, 115, 129, 134], conflict [104, 105, 106, 108], discrimination from family [106, 108, 115, 131], and witnessing or experiencing physical, verbal, sexual, psychological, and emotional abuse [10, 72, 105, 106, 110, 111, 118, 121, 127, 131], including emotional volatility [106, 113, 135]. Others spoke of *academic pressure* [72, 104, 115, 122, 129].

For those with marginalised identities, such as being non‐binary, transgender [106, 131], women [117, 120, 125], autistic [113, 122] and/or racialised [110], these experiences could exist and be amplified within wider sociocultural inequality, such as ongoing exposure to discrimination and victimisation [78, 117, 119, 120, 125, 131]. This was complicated by discrimination towards self‐harm in and of itself (see later sections) [131]. One person with LE in the study by Troya et al. (2019), highlighted how stigma was doubly amplified amongst racialised communities:

Black communities don't talk about these things. This whole conversation wouldn't be happening in a Black or Asian family. It's the decency, you've gotta keep decency. So it's hard for them to get out because the family and community don't want them out.

Such experiences hinted at sociocultural expectations for ‘normativity’ [78, 106, 125, 131], to ‘*live up to*’ [120] expectations [78, 108, 120, 125, 126, 133].

People with LE also explored negative experiences in healthcare [110], such as discrimination, judgement and misunderstanding [78, 110, 111], including clinicians being unknowledgeable about autism and gender diversity [106, 122, 134]. For example, some experienced clinicians doubting they were autistic: ‘‘*Ah, like you are sure? But you don't look autistic’* [109, 134].

Overall, many people with LE explored how others *lacked understanding, support, acceptance and care* [10, 72, 105, 106, 118, 125]. In the next section, we will present how such contexts worked in entangled ways to ‘*act upon’* [31] self‐experience, inducing self‐harm.

#### 3.3.2 Sub‐Theme 2: Embodied Sensations and Self‐Perceptions

This sub‐theme explores the embodied sensations and self‐perceptions people with LE described that were seen as flowing through self‐harm.

Many people with LE described *turmoil and tension* [72, 104, 105, 106, 110, 112, 113, 118, 119, 120, 125, 126], as though ‘*everything is just chaos*’ [118], that was diffuse, overwhelming and enmeshed with self‐perceptions, thoughts, feelings and needs [112, 113, 118], that felt *alien and incomprehensible* [106, 109, 111, 113, 118, 124, 131]. Such experiences felt *incommunicable* [111, 112, 120, 131], and many found it difficult to describe subjectivity [10, 16, 104, 105, 106, 109, 118, 124, 127]. However, visual imagery, physical appearance, songs and metaphors were identified as alternative self‐expression [10, 109, 125, 126]:

…like you get given a pair of shoes, like girls get one pair and boys get one pair (…) And I feel like my pair of shoes, like they didn't really fit and they gave me blisters and they were uncomfortable… [106]

Nevertheless, shame, self‐blame, ‐objectification, worthlessness, disconnectedness, powerlessness, anger, and a sense of deserving harm was explored through relational trauma [16, 105, 107, 108, 118, 120, 125, 128] and/or social structural norms [106, 109, 110, 113, 117, 123, 124]. These could appear seemingly entangled, and it was though people with LE felt misaligned with others, *different* [72, 105, 106, 118, 123, 134], and thus, worthless and bad [10, 72, 110, 119, 120, 125, 131]. For example, an autistic person stated:

I already felt bad about myself and like the abuse I just felt more like a bad person and … I think like that made me vulnerable to be groomed in the first place cause I was like an outsider and like the weird kid so I was like really vulnerable. [122]

For some non‐binary people, disconnection from and discordance with one's body was mediated by the ‘*pain of living in a cisnormative world'* [106]. Similarly, Gunnarsson explored how gendered objectification linked with feelings that ‘*I inhabit this body, but (…) who I am is embedded in the idea of how others see me*’ [119]. Another person felt powerless in experiencing sexual assault, where ‘*it almost felt like a ball inside of me that I just couldn't get rid of’*, that was compounded by having no agency in the criminal process, where ‘*people just didn't get back to me’* [72].

Many reported *self‐directed negativity*, such as self‐hatred, guilt and failure [10, 72, 105, 106, 107, 113, 115, 116, 118, 122, 133]. This also included feeling shame for bad thoughts, ‘*intense emotionality’* and unacceptable feelings [10, 103, 107, 110, 111, 116, 118, 119, 120, 125, 126, 127]:

I had so many times where I was like, I need to cut because I need to, I can't stop the evil. [10]

In ‘*not fitting in*’ [122] with others [134], there was a tension between wanting to be invulnerable and independent [10, 72, 114, 115, 118, 122, 134] whilst also wishing to be seen and heard as oneself [72, 108, 110, 118, 119, 125, 127, 131, 132, 133]. However, some felt they should change to belong [106]:

I want to be accepted, and I think, to be accepted I have to kind of fit into what other people deem to be acceptable … I just feel if [others] knew what I was like they would hate me. [133].

Over time, all of this may all become ‘*too much’*, leading to a *build‐up* [10, 16, 66, 72, 103, 106, 111, 112, 116, 118, 121, 124, 127]:

‘I just get like this build up in my body, I can't really explain it, and it feels like my body's on fire and I get really hot I'm panicky and it's almost like I don't really know where I am’ [122].

Subsequently, people with LE may feel they need to do [112] something to get rid of: ‘… *all these hard feelings*’ [125, 133]. This leads us to self‐harm, and the next section will explore its meanings.

#### 3.3.3 Sub‐Theme 3: Enacting an Embodied Practice

This sub‐theme explores what it means to self‐harm. We present self‐harm as an embodied emotional practice, mediated by social life [16, 117, 119, 127], that engages with corporeal discomfort [16, 117, 127]. It is a private [78, 125, 131], *and* a social practice [125], for ‘*we are extremely aware of how others view us*’ [125], and our ‘*bodies (…) exist within complex spatial and interpersonal contexts’* [117].

Self‐harm method, intensity and frequency changed over time, where it appeared subject to a *push‐and‐pull* [72, 106, 110, 123, 132], or ‘*on and off*’ [110], where people felt in control but then experienced a ‘*spiralling’* [132]. We identified multiple meanings behind self‐harm, consistent with other research. For example, self‐harm alleviated emotions when they were ‘t*oo much’* [10, 16, 72, 104, 105, 106, 107, 108, 109, 110, 112, 116, 118, 124, 127]:

Self‐harm is like a full stop, like punctuation (…) to moods or emotions or to a series of memories. [10].

By doing so, it provided release [10, 16, 66, 72, 103, 104, 106, 110, 111, 112, 118, 121, 123, 125, 127], allowing ‘*control’* over oneself, one's body, emotions and situations [16, 107, 110]. Indeed, one researcher with LE asserted that self‐harm ‘*discipline[s] the self‐body (…) as a form of social control*’ [119], achieving (self‐)mastery [10, 16, 72, 105, 106, 107, 110, 112, 116, 117, 118, 124, 127, 128]. In providing a *full stop*, self‐harm allows people with LE to *‘get on, now you move*’ [136], and assuage suicidality [104, 112, 136]. Thus, self‐harm allowed survival [72, 104, 117] and a reclaiming of power [78, 110, 117, 119]: ‘*Successfully cutting or creating a scar (…) it's powerful*’ [110]. It therefore resisted oppression, such as gender inequity: ‘*I choose what to do with [my] body*’ [119] or restriction in psychiatric institutions:

It probably was a bit of pride, the fact that I took control of things and it was two fingers to the nurses. [72].

Self‐harm also *cultivates and elicits*, transforming ‘*deep aching’ that ‘[burns] a hole right through my body*’ [127] into *real and concrete* physicality [10, 16, 72, 105, 106, 107, 113, 123, 124, 125, 127] to mirror, distract from, understand and substantiate ‘*unseen and inexpressible*’ [124] emotions [10, 16, 72, 104, 105, 106, 107, 118, 124, 127, 128]. For some, self‐harm was ‘*self‐creation in a physical attempt to retell complex, fragmented stories*’ [127], related to ‘*the current situation but also stems from experiences in the past*’ [120], ‘*tell[ing] the story of myself*’ [116, 118, 119, 128], allowing self‐definition [119]. Similarly, self‐harm elicited felt meaning [128] of being alive [113, 119] when feeling ‘*numb’* [16, 104, 105, 107, 112, 116, 121, 123].

I remember sitting in class (…) thinking that I needed to hurt myself to like feel and prove that I was real… [113]

Consequently, self‐harm allowed investigation of and (re)connection with, oneself and one's body [120, 121], to uncover oneself ‘beneath’ and through the skin [113, 127]. One researcher with LE explored how self‐harm upheld social connection by mitigating self‐shame [120, 125], allowing self‐preservation by hiding one's ‘internal’ self [10, 118], maintaining a ‘*hardy persona’* [10, 106]. Thus, self‐harm (re‐)established relational boundaries [118, 119, 120, 125, 126], allowing reintegration within social normativity [106, 119, 120, 126], countering relational disconnection [118, 125, 127]. However, in being unacceptable, self‐harm also (re‐)triggered shame: ‘*I cut again I feel like a psycho, but if I don't cut, I still feel like a psych, ‘cos I want to cut’* [133].

Ultimately, many people with LE wished *to be helped, heard and seen* [72, 106, 110, 119, 120, 125, 127]:

…I felt like everyone was just blanking me, because I'd messed up, so it just added to the shame really (…) in the back of my mind, I was hoping someone would come up to my flat and check on me, and sort of save me, and no one did. [133]

However, if not receiving recognition, self‐harm was *something to rely upon* because ‘[*it] gives you that comfort that you're not getting from other[s]*’ [110], representing a symbolic healing [103, 117, 119, 121, 124, 127, 131]. Self‐harm was also a way to *direct negativity to oneself*, where some felt they ‘*deserved it* [106, 115, 132]’ or needed to express anger [72, 105, 106, 107, 112, 119, 120, 123, 127], where directing it at oneself felt ‘*safer’* [72, 105, 113, 118, 133] and more socially acceptable [123]. In the next section we explore LE perspectives of self‐harm, and their experiences of others' responses.

#### 3.3.4 Sub‐Theme 4: People's Relationships to Self‐Harm

This sub‐theme describes what people with LE thought of self‐harm and their experiences of others' perspectives, including family, friends, peers and healthcare professionals.

Many people with LE may want to stop self‐harm [104]. However, it was also transformative [16, 116], an ‘*emotional ‘crutch'*’ [105] that superseded alternative methods [103, 104, 105, 112, 125]. One person with LE described their ‘*intimate relationship*’ with ‘it’*:*

… I do think it is something that I know so intimately, and it knows me (…) I have a relationship with it that I don't have with anyone that I know in person, and no‐one will know that relationship either, which I think keeps it being such an intimate thing. [136]

Others constructed self‐harm, including scars, as a part of oneself, one's life, identity and story [78, 109, 118, 119, 125, 126]. Others *rationalised engagement* [10, 16, 79, 112, 117, 119, 124], positioning it as ‘*valid’* [78], and ‘*normal’* [121]. Some drew upon biomedical understanding, such as release of ‘*pain‐relieving ‘chemicals*’ [16, 111], or framed it as *addictive* [111] because it involved strong ‘*mental and physical urges’* [104], that meant ‘*you don't want to stop’* [136]. Simultaneously, self‐harm was *harmful and bad*, where it does not always ‘*work’* [105], should not be normalised [119], and could exacerbate *shame* and *guilt* [113, 114, 133, 136].

People with LE were conscious of social constructions of self‐harm as ‘bad’ [113], where it indicated a lack of capability or mental stability [129] because ‘*normal people don't do this’* [114]. Further, awareness of the ‘*typical self‐harmer’* meant some were conscious if they did not fit this profile [129, 136]. Others anticipated the *gaze of others*, worrying about upsetting, angering, shaming or disappointing others [113, 114, 132, 133]. People with LE were also met with dismissal, judgement and discrimination [106, 132, 133]: ‘… *[she said], everyone's embarrassed by you … you need to cover up [the scars on] your arms’* [113, 133].

Some described clinicians being dismissive [106, 132, 133], paternalistic, having ‘*unhelpful, insincere or uncaring’* attitudes [78], blocking access to care, and escalating distress [132]. Others were met with *silence*: ‘*I had one boyfriend who did notice (…) but he didn't ask so I just never said anything…’* [114]. Such responses meant some learned that self‐harm was ‘*not something people want to know’* [114] about, and so *hid themselves further* by lying about and concealing self‐harm, including changing where they self‐harmed and how they spoke about it [108, 110, 113, 129, 136]: ‘*I almost feel like I have to say the right thing for the therapist’* [134].

Ultimately, it appeared then that people with LE felt at fault [108, 113, 114], amplifying shame [105, 108, 113, 136], where ‘*you feel crap every time you look at yourself, every time you see them scars’* [105], with others exploring how this prompted further self‐harm [103, 104, 105, 113, 133].

## 4 Discussion

### 4.1 Summary of Main Findings

Through undertaking this IR within a survivor, participatory approach, we argue that social and relational harm moulds the bodies of people with LE leading to embodied sensations of tension, self‐hatred and self‐perceptions of oneself as wrong, bad and different to others. In feeling they are worthless, people with LE may then understandably feel they ‘*deserve’* to self‐harm to be acceptable to oneself, to others and the world. Thus, we highlight how self‐harm speaks to, as explored by Heney (2020), the messy, uncertain complexity of what it means to be a body in social life [116, 117, 127]. Consequently, our work continues to challenge the hegemonic biomedical model that relegates self‐harm to individual responsibility [19, 21, 24, 103].

In the following section, we build upon these ideas, exploring how psy‐knowledge is consistent with, and leaks into [31], wider societal constructs that centralises people with LE as responsible for how they feel about what they experience and how they negotiate with this [127]. Thus, we suggest that it is not only sanism outside of psychiatry that contributes to people's distress, but that psy‐culture in and of itself (re‐)produces notions of social normativity, compounding distress. Our work is inspired by Inckle, Chandler, Brossard and Steggals [1, 28, 31, 102], and builds upon burgeoning survivor work [24, 37, 65, 72, 137, 138], to continue to draw ‘*attention to the real causes of distress’* [27], where ‘personal troubles’ are moulded with(in) sociocultural life [19, 29, 31, 91].

### 4.2 The Liminality of Self‐Harm: Individual ‘Problem’ or Embodied Social Distress?

This section will first critically explore how researchers variably constructed self‐harm (**Theme 1**). We then move onto to discussing **Theme 2**, where we consider how ‘inner’ embodied sensations appear to ‘mirror’ the complexities of ‘outer’ social life. This section is denoted ‘*The Liminality of Self‐Harm* 1’ to describe how self‐harm shows, as explored by Chandler, ‘*the body as both ‘actor’ and ‘acted’ upon*’ [31]. Findings were explored amongst CdCL, SB, GJ and EM, and are informed by our interpretations.

**Figure 4:**
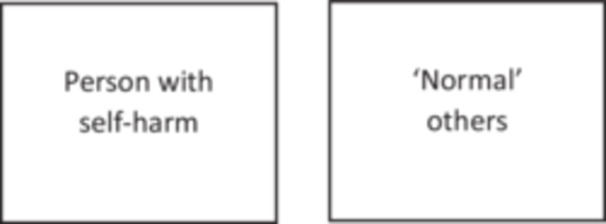
Clinical construction of the person with self‐harm, inspired by Turp [75].

Sociologists with LE discussed how self‐harm is situated as a pathological behaviour that manages ‘negative emotionality’. Thus, self‐harm becomes ‘boxed up’ within static categories: it ‘*is’* a ‘maladaptive’ behaviour: it is ‘bad’, not ‘good’. This formulaic categorisation hints at, often implicit, adherence to positivism [2] and either/or binaries across psychological and psychiatric understanding [2]. Indeed, LEAP members considered psy‐knowledge as ‘*black and white*’, that feeds into societal ideas of ‘acceptable’ versus ‘unacceptable’ behaviours [127]. Thus, we argue that self‐harm is individualised and problematised as a distinct category seemingly separate from social life. This was denoted by CdCL as ***a divided binary*** [16, 78, 120] (see Figure 4).

Language is not neutral and the ways we discuss human experiences are linguistic choices that shape our perceptions [139]. As Giacomini notes: ‘*Semantics deeply entangle values with fact, because language mediates the expression of facts. The words that describe research (…) carry ethical valence*’ [48]. Thus, through clinical constructions, we argue, self‐harm factually *becomes* an ‘individual problem’, where the person is [78] the container for ‘*aversive emotionality’*. This obscures and distorts what it means to be a body existing within social life: that we, and by extension self‐harm, do not exist as a silo independent of relational and societal discrimination, trauma and abuse, and ‘*emotional dysregulation’* underpinning self‐harm may be a valid, resilient response to oppression and violence [47, 140]. However, as psy‐knowledge carries social authority, it continues to be prioritised as ‘*the truth*’, rather than ‘*a truth’*, little room is left for alternative thinking [26, 80, 81]. Thus, self‐harm arises from the ‘‘*inner’ and therefore not ‘outer*’ [141], and gets, as the LEAP noted, ‘*put in a bubble’*, and people's experiences fade into the background [19, 75, 142].

Our findings highlight how variable reactions from others, including clinicians, can amplify shame and alienation, leading to concealment and further engagement. We extend such ideas to explore how psy‐knowledge itself may underpin such reactions, because, as we discuss, within a psychiatric/psychological framing, self‐harm *is* an unhealthy behaviour and thus, needs to be prevented, discouraged or stopped. If the individual does not ‘cease’, and/or choose ‘healthier’ alternatives, *they* are solely responsible (LEAP members) [138, 143].

**Figure 5:**
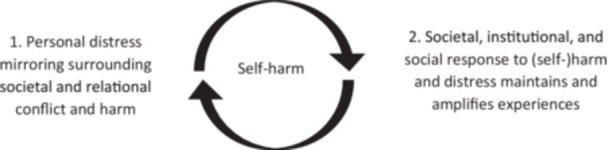
A self‐fulfilling prophecy: The origin, amplification, and maintenance of self‐harm, inspired by Gunnarsson and informed by LEAP expertise [118].

Therefore, whilst not endorsed by NICE guidelines [4], in practice, we see this play out as the rationalisation of unhelpful clinical assumptions, ‘no engagement’ self‐harm contracts during treatment contact, restrictive practice, and withholding care and communication to dissuade or stop self‐harm [22, 110, 138, 144]. This is shown in recent research: Webb et al. (2025) found people with LE currently experiencing domestic abuse and self‐medicating were denied service access because ‘*mental health services appeared to reject any complexity and were more interested in categorising*’ and people with LE were ‘*thrown in a box*’ [66]. Likewise, in our review, one person was dismissed because they ‘*just needed to keep* themselves *safe*’ [109, 132]. Thus, people with LE may feel and be aware that many may think: ‘*Why can't* you *just be fine?’* [24] (our emphasis). Subsequently, psy‐knowledge, as intertwined with societal notions of normativity, that underpins attitudes and responses to self‐harm, may not only misunderstand people's LE but (re‐)establishes them against a negative construct [82]: as to blame and different from ‘normal’ others [22, 27, 77, 145, 146]. We argue that this ‘*taps into’* existing distress originating from other social and relational harm, working as a self‐perpetuating [120], or ‐fulfilling prophecy (Figure 5), for, as EM noted: ‘*If you are told you are bad, that is what you become*’ [82].

Psy‐understanding and responses may also implicate *how* people with LE speak about self‐harm. In this review, CdCL interpreted many with LE wanting to be recognised by others, sometimes through self‐harm [147], and sought to present themselves as ‘acceptable’ [134]. Thus, whilst many may endorse clinical conceptualisations [27], it may be that reliance on this makes speaking about self‐harm more accessible to oneself, *and* others, increasing credibility and reducing risk of minimisation [34, 77]. Indeed, Hill & Dallos (2012) suggested that adolescents’ exploration of self‐harm appeared influenced by clinical explanations, where ‘‘*neat’ superficially credible descriptions*’ [148] masked complexity. This could hint at hermeneutical injustice, in which gaps ‘*in collective interpretative resources puts someone at an unfair disadvantage when it comes to making sense of … social experiences*’ [149]. In other words, someone with LE may not ‘*communicate [their] own suffering because the words for it do not yet exist.*’

Furthermore, other research highlights how sharing of trauma, abuse and other difficult experience is constrained by fears of being disbelieved, unheard or subject to unhelpful assumptions, including being judged for self‐harming as a response [65]. Indeed, this response is likely for as self‐harm activist Clare Shaw noted after sharing her experiences of childhood abuse, clinicians replied: ‘*So for all those years, did you not try to tell anyone what was going on for you?’* [27]. This testimonial injustice is compounded by the risk of being delegitimised if LE perspectives do not match wider psychiatric and societal conceptualisations [12, 15, 34, 115]. As LEAP members noted: ‘*You have relationships both with self‐harm and the discourse around it’*, and this works to implicate self‐experience, and the sharing of oneself, in complex, embroiled ways. Therefore, it is important to make space with which people with LE can adhere to the right language for *them*, not for us as clinicians, to feel embodied (self‐)acceptance, recognition and validation [27]. As the non‐binary writer Jespa Jacob Smith noted when discovering they/them pronouns: *‘… for the first time, just by chance, language had finally managed to manifest myself in my body’* [150].

In summarising this section, we conclude psy‐knowledge fails to be accountable to how it may (re‐)enact distress by blaming individuals as culpable, whilst obscuring its embedment within social norms [11, 77], losing sight of ‘*complex webs of social relationality*’ [117]. As one writer with LE notes: ‘*They are obsessed with the labels they can put on me rather than ever looking inwards at themselves*’ [39]. However, as Shaw notes, *‘…understanding and working helpfully with self‐injury begins with the self*’ [26].

In moving onto **Theme 2**, we see that many with LE explore past and ongoing relational and social harm, societal inequality and expectations for ‘normativity’. As Malson (2023) suggests ‘*society inhabits every individual’*, and so, relational interactions between self and other speak of the wider world, and each weave into one another [127]. This is important to attend to because psy‐knowledge devolves messy complexity into categories [131], and so, trauma may be treated as discrete events relegated to the past [65], and the survivor is typically centralised, such that how *others* perpetuate harm *onto* them is silenced [11].

Alternatively, as discussed in trauma research, dismantling experience, even if historical, has ‘*no absolute boundary between here‐now and there‐then*’ [151], making it difficult to name and know one's emotions in the present [16, 139, 152]. Other research centralises how abuse of power is inherent to trauma, including societal inequality, where one has power over the other, distorting clarity and minimising one's sense of self [153]. It is perhaps not surprising then that people with LE describe chaos; embodied distress, tension and discomfort, and an internalised vocality, as ‘bad’ thoughts, that may be obscure but also, replicate social experiences in understandable ways [148]: ‘*It's [anorexia nervosa voice] almost like that (…) angry parent, angry bully*’ [66, 154], that feel like ‘*everything all at once’* [146, 155]. Thus, ‘inner’ sensations are not just individual, discrete entities but what Brossard notes as ‘*social tension’* expressed through the self‐body [156]. Treating this as ‘*stress’* risks framing epistemic violence, like racial abuse, as the anti‐racist scholar Kendi notes, as a ‘*passing inconvenience*’ [157].

**Figure 6:**
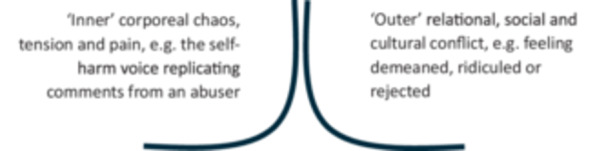
Trajectory: ‘Inner’ bodily chaos reflecting ‘outer’ social experience, inspired by Gunnarsson, Brossard, Ahmed and Kelland [27, 78, 150] and LEAP expertise.

It was common for people with LE to report that self‐harm serves to resolve escalating chaos ‘*to avoid a catastrophic eruption*’ [30], as consistent with other self‐harm research However, this does not reach an ‘*end point’*, as noted by LEAP members, rather self‐harm involves ‘*fluidity’*, which CdCL named ***a dynamic flow***, where the phenomenon is not just a behaviour but a ‘*vehicle’* that moves through one's past, present and future [119], ‘*trajectory*’, as noted by SB, and a ‘*complex changing relationship in flux between good and bad’* (LEAP members) (see Figure 6).

Therefore, self‐harm uncovers a ‘*false dualism*’ [78], occurring across body/mind, ‘inner’/‘outer’, agency/powerlessness and destruction/care [110, 116, 117, 120, 121, 124, 126, 127, 128, 158], where self‐harm negotiates with ‘*the aggregated consequences of social issues’* [28]. This *liminality* is summarised by one sociologist:

I locate (…) self‐harm as an experience in which I was exercising control over myself, my body, and my life, but also as something that happened to me, which at times felt (…) beyond my ability to even comprehend. [117]

Thus, self‐harm can be conceptualised as an embodied practice that negotiates with and responds ‘*to the self and the self's place in the world’*.

## 5 Strengths and Limitations

### 5.1 Quality of Included Articles

Included studies had moderate to high CASP score (ranging from 14 to 19). Rationale for research was extensively explored, with authors justifying the qualitative approach clearly and some considering means of enhancing accessibility. Despite scoring moderately or highly in most CASP questions, many studies failed to sufficiently explore researcher/participant relationships, reflexivity and ethics. Thus, the majority scored ‘0’ or ‘1’ for questions 6 and 7 respectively [159]. Indeed, it was common that researchers stated identities without exploration, demonstrating how reflexivity is limited by insufficient self‐engagement [160, 161]. As Enloe (2016) notes, reflexivity ‘*should never be comfortable or easy*’ and should be ‘*less about oneself and instead should be focused on humility and practice‐oriented disruption of unethical research’* [162]. Without this, reflexivity is unlikely to achieve more equitable research practice but rather makes us ‘*more aware of asymmetrical or exploitative relationships’* [162], whilst re‐centring the researcher who holds the power.

Regarding PPI, 22 articles were led by, or co‐authored with, researchers with LE of which three also collaborated with an advisory panel, who advised on interview schedules [115, 163] or throughout [133]. The remaining fifteen solely had an advisory panel, two of whom consulted onthe project throughout or advised on certain aspects, such as the protocol, research materials or data analysis. Most studies did not discuss PPI thoroughly, making it unclear as to whether there was authentic inclusion. In two studies [107, 111], advisory groups included both researchers and people with LE, but authors did not explore how power differentials may have impacted space safety [47]. Some researchers disclosed LE. However, only three also collaborated with others [115, 133, 163]. As discussed, solely relying upon one LE voice in research does not enhance inclusivity. To maximise opportunities to do so, all researchers should build relationships with others with LE and transparently report this, including explicitly interrogating their own position [85, 89].

EDI principles were reflected upon by CdCL to explore whether research prioritised community voice. CdCL provides partial insight into LE and trauma‐informed approaches. However, to also strive towards self‐accountability, ‐awareness and challenge their complicity in white racial privilege, CdCL attends, and will continue to attend, decolonialisation and anti‐racism education, engage with anti‐racism and anti‐oppressive literature [157, 162, 164, 165] and others’ accounts of LE across intersectional identities [23, 27]. This is with the hope of working towards ongoing white reflexivity [70].

Some studies acknowledged how marginalised voices are excluded but few made steps towards mitigating this [16, 106, 123, 124]. Indeed, samples were mostly young, white British, cisgender women, aligned with stereotypes of the ‘*typical self‐harmer*’ [102]. The repeat search in Apr‐26 gleaned a significant number of UK articles exploring self‐harm in autistic people and those identifying as LGBTQ+: a positive move towards inclusivity. However, these included a significant proportion of only white participants, without acknowledgement or critical exploration, demonstrating the continuing, but often unspoken, prioritisation of white voices in mental health research [167,168]. Three studies used creative‐based methods – collaborative art making and photo‐elicitation [10, 123] potentially promoting multivocal engagement and accessibility [47].

### 5.2 Methodological Critiques of Current Study

All spaces, including those claiming to be anti‐oppressive, are subject to (re‐)perpetuating systemic inequality [12, 45]. Indeed, survivor spaces, as with research institutions, are dominated by white researchers, excluding people of colour [12, 26] and through ‘LE’, as explored by Voronka (2016), risk implying a unified category, negating heterogeneity. To work against oppressive practice, acknowledging we too are embedded within psy‐disciplines [48, 58], we have sought to locate ourselves outside an epistemological frame that begins with the individual as a ‘problem’ [58]. Further, we consider EDI principles and employ reflexivity to work towards greater solidarity and self‐reflection. We acknowledge this complexity and we wish to remain accountable to this ongoingly to mitigate against complacency.

Likewise, whilst PPI aims for accountability to people with LE, often, they are only ‘offered a seat at the table’, where ‘*consultation exercises (…) most likely aren't brought in right at the start of an idea and given equal share of the resources and responsibility*’ [88]. There are ways in which this was also apparent in our own work. For example, equitable power sharing was restricted by limited resources and this work forming part of CdCL's doctorate. Peer authors were given regular opportunities to edit the manuscript, but CdCL developed drafts. Further, peer authors were presented with data summaries, but findings were initially guided by CdCL. As such, research was dictated by CdCL, limiting its emancipatory function.

In our work, a significant limitation that we reflected upon is that by using a review methodology and RTA, we risk presenting a collective summary of self‐harm across identiities, failing to sufficiently employ intersectionality. Thus, our work risks homogenising and obscuring unique experiences across marginalised identities, particularly those who are racialised and subject to complex, enmeshed, intersecting systems of oppression, such as women of colour [101]. Future research could use alternative methods that can acknowledge and explore how LE of self‐harm is shaped by the intersect of one's identities, sociocultural and political positioning [12].

Further limitations relate to a lack of double screening, and the quality assessment was conducted by only one researcher. The search strategy is likely constrained because PPI was not always being discussed in the main body of manuscripts and additional terms related to PPI would have constrained the search, limiting reliability. Additionally, the broad age range lessens specificity. Finally, including English only literature is no longer an acceptable criterion. This is a major limitation of our work, excluding the perspectives of the Global Majority, maintaining a Eurocentric lens and limiting transferability by perpetuating a colonial frame. This is compounded by using UK‐centric PPI definitions.

## 6 Research Implications

In conducting a review, it is important to explore what was said, but also, what was not. Firstly, we identify how some voices continue to be dimmed in self‐harm research, such as people who are racialised, men, and gender diverse people, particularly transwomen. Thus, self‐harm continues to be constructed as existing within young white cis‐women and this means those who do not ‘*fit the typical profile’* are silenced [170]. Thus, in future research, underserved voices should be centralised and space given to specifically consider self‐harming experiences across intersecting identities. Second, we highlight how psy‐knowledge continues to individualise and problematise self‐harm. This may harm people with LE, (re)‐producing feelings of blame and failure, and prompting further engagement. Further research investigating LE and psychiatric perspectives is required to elucidate this to consider with greater clarity what harms, but also what heals.

## 7 Conclusion

Through LE perspectives, this review builds upon critical sociological theorisation and survivor work to offer an interdisciplinary understanding of self‐harm as an embodied practice of self‐expression within social life. In extending knowledge in this area, our `work highlights the need for psychiatric practice to consider the complexity of self‐harm, to ensure people with LE feel validated, seen and heard.

## Author Contributions

**C. C. da Cunha Lewin:** conceptualisation, investigation, methodology, formal analysis, project administration, funding acquisition, writing – original draft. **S Baldoza:** conceptualisation, validation, writing – review and editing. **G Jerwood:** conceptualisation, validation, writing – review and editing. **Es Miles:** conceptualisation, validation, writing – review and editing. **M. Leamy:** conceptualisation, supervision, writing – review and editing. **U. Foye:** supervision, writing – review and editing. **A. Sweeney:** conceptualisation, supervision, writing – review and editing.

## Conflicts of Interest

The authors declare no conflicts of interest.

## Supporting information

Supporting File

## Data Availability

The data that support the findings of this study are available in the supporting information of this article.

## Endnotes

1 Naming of this interpretative theme was inspired by Stjernegaard et al. (2025) and Steggals (2016).
