# Supplementary material for: Critically Exploring Self‐Harm Through Lived Experience Perspectives: A Survivor‐Controlled Integrative Review Employing Participatory Methodology
Source: Health Expect. 2026 Jun 7;29(3):e70700. doi: 10.1111/hex.70700 (PMC13242651; doi:10.1111/hex.70700)
Supplement: Supplementary file 1 — Supporting File [file HEX-29-e70700-s001.docx]

**Supplementary Materials
Supplementary Material 1:** Adapted qualitative CASP for quality assessment, where answers quantified to provide overall CASP score where: 0 = No, 1 = Somewhat/can’t tell, 2 = Yes.

| **No** | **Author(s)** | **Q1: Was there a clear statement of the aims of the research?** | **Q2: Is a qualitative methodology appropriate?** | **Q3: Was the research design appropriate to address the aims of the research?** | **Q4: Was the recruitment strategy appropriate to the aims of the research?** | **Q5: Was the data collected in a way that addressed the research issue?** | **Q6: Has the relationship between researcher and participants been considered?** | **Q7: Have the ethical issues been taken into consideration?** | **Q8: Was the data analysis sufficiently rigorous?** | **Q9: Is there a clear statement of findings?** | **Q10: How valuable is the research?** | **Total score (20)** | **Summary of issues** |
| --- | --- | --- | --- | --- | --- | --- | --- | --- | --- | --- | --- | --- | --- |
| 1 | Boyce et al., (2021) | 2 | 2 | 1 | 2 | 1 | 0 | 1 | 1 | 2 | 2 | 16 | No reflexivity and minimal discussion of ethics. Open-ended questionnaire means follow up is not possible (acknowledged as limitation), so meaning constrained. |
| 2 | Brown et al., (2022) | 2 | 2 | 2 | 1 | 2 | 1 | 2 | 1 | 2 | 2 | 17 | Justifications and drawbacks of recruitment not discussed in detail. For example, gatekeeping issue and eating distress exclusion. Reflexivity only involved disclosing identities and stating that they influenced results but not how. Only cited ethics approval, no further discussion. |
| 3 | Chandler & Simopoulou (2021) | 2 | 2 | 2 | 0 | 2 | 2 | 2 | 2 | 2 | 2 | 18 | Participant/researcher relationship and positionality explored, acknowledgement of ethics. Recruitment strategy not stated. |
| 4 | Chandler (2012) | 2 | 2 | 2 | 2 | 2 | 0 | 0 | 2 | 2 | 2 | 16 | No consideration between researcher and participant, minimal ethical considerations |
| 5 | Chandler (2013) | 2 | 2 | 2 | 2 | 2 | 0 | 1 | 2 | 2 | 2 | 17 | No consideration of researcher relationship, minimal ethical considerations |
| 6 | Chandler (2014) | 2 | 2 | 2 | 2 | 2 | 0 | 1 | 2 | 2 | 2 | 17 | No consideration between researcher and participant, minimal ethical considerations |
| 7 | Donskoy & Stevens (2013) | 1 | 2 | 2 | 2 | 2 | 0 | 2 | 2 | 2 | 2 | 17 | No consideration of researcher/participant relationship, unclear research gap |
| 8 | Edmondson et al., (2013) | 2 | 2 | 2 | 2 | 2 | 0 | 0 | 2 | 2 | 2 | 16 | No consideration of researcher/participant relationship, no ethical considerations |
| 9 | Gosling et al., (2023) | 2 | 2 | 2 | 2 | 2 | 1 | 2 | 2 | 2 | 2 | 19 | Minimal consideration of researcher/participant relationship, reflexivity minimal |
| 10 | Gunnarsson (2023) | 2 | 2 | 2 | 0 | 2 | 2 | 2 | 2 | 2 | 2 | 18 | No discussion of recruitment |
| 11 | Jackman et al., (2018) | 2 | 2 | 2 | 2 | 2 | 0 | 0 | 2 | 2 | 2 | 16 | No consideration of researcher/participant relationship or ethics |
| 12 | Lockwood et al., (2021) | 2 | 2 | 1 | 1 | 1 | 0 | 1 | 2 | 2 | 2 | 14 | Methods may be constrictive, implicating the direction of the conversation; minimal to no consideration researcher relationship or ethics |
| 13 | Lundwall et al., (2025) | 2 | 2 | 2 | 2 | 2 | 2 | 1 | 2 | 2 | 2 | 19 | Reflexivity more in depth than other studies, exploring emotional reactions and how these were mitigated in interviewing. Extensive trustworthiness section. |
| 14 | Miller et al., (2021) | 2 | 2 | 2 | 1 | 2 | 0 | 1 | 2 | 2 | 2 | 17 | All-women inclusion limiting applicability and reducing inclusivity, no consideration of researcher/participant relationship |
| 15 | Morris et al., (2015) | 2 | 2 | 2 | 2 | 2 | 0 | 1 | 2 | 2 | 2 | 17 | No consideration of researcher/participant relationship, minimal ethical considerations |
| 16 | Mughal et al., (2023) | 2 | 2 | 2 | 2 | 2 | 0 | 1 | 2 | 2 | 2 | 17 | Minimal ethical considerations, no consideration of researcher relationship |
| 17 | Naz et al., (2021) | 2 | 2 | 2 | 1 | 1 | 0 | 2 | 2 | 2 | 2 | 16 | Exclusion of people with mental distress, suicidality in self-harm for participants, mismatch with chosen definition of self-harm, no consideration of researcher relationship. |
| 18 | Norman et al., (2022) | 2 | 2 | 2 | 1 | 2 | 0 | 2 | 2 | 2 | 2 | 17 | No consideration of researcher/participant relationship, limited recruitment, incentives for participation (university credit) |
| 19 | Norman et al., (2023) | 2 | 2 | 2 | 1 | 2 | 0 | 2 | 2 | 2 | 2 | 17 | No consideration of researcher/participant relationship, limited recruitment, incentives for participation (university credit) |
| 20 | Pollock et al., (2021) | 2 | 2 | 2 | 1 | 2 | 0 | 1 | 2 | 2 | 2 | 16 | Minimal consideration of researcher/participant relationship, limited recruitment avenues. |
| 21 | Rebbettes & Bacon (2025) | 2 | 2 | 2 | 2 | 2 | 1 | 0 | 2 | 2 | 2 | 17 | No discussion of ethics, reflexivity but not explicit explanation of the relationship between participant/researcher. Acknowledges lack of inclusion of autistic peers. |
| 22 | Rosenrot & Lewis (2025) | 2 | 2 | 2 | 1 | 2 | 1 | 0 | 2 | 2 | 2 | 16 | Reflexivity limited, no ethical exploration or discussion of relationship between researcher/researched |
| 23 | Russell et al., (2010) | 1 | 2 | 2 | 2 | 2 | 1 | 2 | 2 | 2 | 2 | 19 | Slightly unclear examination of the aims of research |
| 24 | Sabo et al., (2025) | 2 | 2 | 2 | 1 | 2 | 0 | 0 | 2 | 2 | 2 | 15 | No discussion of gatekeeping risk, reflexivity was limited, statements of identity, nothing about interview rapport etc., |
| 25 | Stanicke (2021) | 2 | 2 | 2 | 1 | 2 | 1 | 0 | 2 | 2 | 2 | 16 | Minimal consideration of researcher/participant relationship, no ethical considerations |
| 26 | Troya et al., (2019) | 2 | 2 | 2 | 2 | 2 | 0 | 2 | 2 | 2 | 2 | 18 | No consideration of researcher/participant relationship |
| 27 | Williams et al., (2023) | 2 | 2 | 2 | 2 | 2 | 2 | 1 | 2 | 2 | 2 | 19 | Reflexivity and rapport in interviews discussed in detail in supplementary materials, no discussion of ethics. |
| 28 | Witcher et al., (2025) | 2 | 2 | 1 | 2 | 2 | 2 | 2 | 2 | 2 | 1 | 18 | Audio vignettes seem a risk in potentially swaying participants, one pilot interview with one EbE does not necessarily mean people in interviews would not feel it is socially desirable to agree with the researcher. Situating self-harm as an addiction may reinstate it within an illness discourse. Some reflexivity. |
| 29 | Woodley et al., (2021) | 2 | 2 | 2 | 2 | 2 | 0 | 2 | 2 | 2 | 2 | 18 | No consideration of researcher/participant relationship |

*Reflexive critical essays not included for CASP assessment

**Supplementary Material 2:** Adapted Guidance for Reporting Involvement of Patients and the Public (GRIPP2-SF)***.**

| **No** | **Author(s)** | **Section 1 - Aim(s) of PPI**  *Was the aim of PPI reported in the study?* | **Section 2 - Method(s)**  *Was there a clear description of the methods used for PPI in the study?* | **Section 3 - Study results**  *Were the results of PPI in the study, including both positive and negative outcomes reported?* | **Section 4 - Discussion and conclusions.** *Was the extent to which PPI influenced the study reported overall? Including the positive and negative effects?* | **Section 5 - Reflections and critical perspectives.** *Were there critical comments on the study, with reflections on the things that went well and those that did not, to inform others’ experiences?* | **Comment** |
| --- | --- | --- | --- | --- | --- | --- | --- |
| 1 | Boyce (2017) | No | No | No | No | No | Author contacted separately, worked with an advisory panel, only some had self-harm. They reviewed proposed study design, PIS and survey questions. |
| 2 | Brown et al., (2022) | No | No | No | No | No | Author contacted separately, LE consultant as co-author |
| 3 | Edmondson et al., (2013) | No | No | No | No | No | Service-users involved in the study design only (p10) in acknowledgements. |
| 4 | Gosling et al., (2023) | No | Somewhat | No | No | No | Experts by Experience consultation group had input into interview schedule (p345). |
| 5 | Jackman et al., (2018) | No | No | No | No | No | Advisory board commented on “all aspects of the study” (p18) as per the acknowledgments, not discussed in main article, author contacted separately, not all people on the board had an experience of self-harm. |
| 6 | Lockwood et al., (2021) | No | No | No | No | No | Not discussed in article, author contacted separately: advisory group contributed to study process, measures and card-sort task. |
| 7 | Lundwall et al., (2025) | Yes | Yes | Yes | Yes | Yes | Involved two external ‘insider’ advisors in data analysis process, included ways they impacted and influenced results, with section at the end of results to explore their impact. |
| 8 | Miller et al., (2021) | No | Somewhat | No | No | Somewhat | PPI members contributed to study design, with some feedback from members (p4). |
| 9 | Morris et al., (2015) | No | Somewhat | No | No | No | Briefly mentioned methods (p127). |
| 10 | Mughal et al., (2023) | Somewhat | Yes | No | Somewhat | No | PPI advisory group contributed to study conception, design and interpretations (p1163). However, advisory group consisted of public members and support workers. |
| 11 | Naz et al., (2021) | No | No | No | No | No | The authors acknowledged their PPI group only (p10). |
| 12 | Norman et al., (2022) | No | No | No | No | No | Author contacted separately to determine PPI, not discussed in article. |
| 13 | Norman et al., (2023) | No | No | No | No | No | Author contacted separately to determine PPI, not discussed in article. |
| 14 | Pollock et al., (2021) | No | Somewhat | No | No | No | One young person with LE consulted on the semi-structured interview guide. |
| 15 | Russell et al., (2010) | No | Somewhat | Yes | Yes | No | Brief information provided as to how PPI was achieved, with guidance provided by people with LE included in article. |
| 16 | Stänicke (2021) | Yes | Somewhat | Somewhat | No | No | Importance of PPI and its method briefly discussed (p4). |
| 17 | Troya et al., (2019) | Yes | Yes | Yes | Yes | Yes | Detailed description of PPI in separate published article. |
| 18 | Williams et al., (2023) | No | Yes | No | No | No | LE-led with input from an advisory group LGBQT+ young individuals who had experience of SIB. Authors mentioned piloting with two individuals, implicitly suggesting this was also involvement, however, pilot interviews are not PPI. |
| 19 | Witcher et al., (2025) | No | Yes | Somewhat | No | No | LE-led with input from an advisory group, throughout the research lifecycle. Mentions a pilot interview with an EbE, not PPI. |
| 20 | Woodley et al., (2021) | No | Somewhat | No | No | No | Author contacted separately, no discussion in article, co-authored with LE collaborator. |

**Supplementary Material 3:** Adapted tool for critically reflecting upon the Equity, Diversity and Inclusivity of studies, with influence from survivor ethics and trauma-informed approaches

| **No** | **Author(s), Year** | **1. Approach to embedding diversity and inclusion in the research lifecycle.** *Is EDI embedded during the planning of all activities to remove as many barriers as possible?* | **2. Good practices in recruitment and/or selection processes to ensure diversity.** *Have all opportunities been openly advertised through diverse channels considering language, flexibility and reasoning for criteria? Have people been fairly recruited, which incorporates current good practice? Has consideration been given to positive action whilst avoiding overburdening and creating an imbalance of expertise?* | **3. Inclusive and accessible environment.** *Has an inclusive environment been established where all can thrive, and all voices are valued? Was regular communication provided?* | **4. Inclusive research.**  *Is diversity included in the research design? For example, is the user voice included in the research and/or consideration is given to ensuring diversity in study participants. Are research outputs accessible and inclusive?* |
| --- | --- | --- | --- | --- | --- |
| **1** | Boyce (2017) | Not explicitly explored but Boyce discusses the misconceptions and assumptions about self-harm only appearing in adolescence, considering how research focuses on young people. Mostly women in participant, unclear whether included those from racialised communities, some information related to current job situation of which 5 (out of 19) were unemployed. | Uses an online community forum to recruit participants, not diverse recruitment, justifies use of online forum recruitment. | This was not discussed | As mentioned, focusing on underserved group (as Gunnarsson explores in their research, ageism often affects those who self-harm who are middle-aged or older). A service user research group of whom some had LE of self-harm reviewed the proposed study design, PIS and survey questions, some of whom had lived experience of self-harm. |
| **2** | Brown et al., (2022) | This was not discussed. | Gatekeeping from clinicians not discussed, but option for participants to self-refer. Exclusion of people with eating distress, but later issue as participants themselves speak of eating distress as self-harm | Issue of English language criterion, no longer used due to exclusion risk. Used people’s own words to structure interview. | Involvement of LE collaborator as co-author but detail not given, inclusive of those from racialised communities. |
| **3** | Chandler & Simopoulou (2021) | The researchers discussed the continuing emphasis of self-harm research on young White women, despite the appearance of self-harm in men and across the lifespan. The researchers acknowledged the lack of self-harm research examining race and ethnicity. | This was not discussed. | The researchers used a novel method that combined group discussions and art making. They did so to allow for flexibility in exploring the nuance of self-harm experiences and making space for creativity and innovation. Employing this creative methodology may serve to maximise accessibility and engagement. | One of the researchers had a LE of self-harm, centring user voice. Socio-demographic information characterising the sample, such as socioeconomic background, race and ethnicity, sexual orientation and gender were not discussed. |
| **4** | Chandler (2012) | The impact of clinical biases leading to research focusing on certain groups, such as women, young people and clinical patients was briefly noted. | This was not discussed. | This was not discussed. | The research was led by a person with LE, but no other means of trying to include voices occurred. Recruitment had equal numbers of men and women. Sample was noted as relatively diverse, with mixed socio-economic backgrounds and current employment status, variable self-harm methods and experiences with formal services. |
| **5** | Chandler (2013) | The focus of self-harm research on women, young people and psychiatric or hospital patients was briefly noted. | The recruitment strategy was designed to maximise inclusion of a varied sample, with an emphasis on those with little or no engagement with formal medical treatment, older age adults and equal numbers of men and women. The advertising channels were not specified. | This was not discussed. | The research was led by a person with LE. The sample was characterised as somewhat diverse: most were undertaking undergraduate degrees, but there was variation in socio-demographic backgrounds, and included men. 10 participants were British White; ethnicity of remaining 2 was not given. Other sociodemographic information relating to socioeconomic background, race and ethnicity, sexual orientation and gender were not discussed. |
| **6** | Chandler (2014) | This was not discussed. | This was not discussed. | This was not discussed. | The research was led by a person with LE. The sample was described according to age, gender, self-harm method, presentation and engagement without mention of other socio-demographic characteristics. |
| **7** | Donskoy & Stevens (2013) | The researchers briefly acknowledged that men are given less opportunity to take part in research. | The recruitment strategy was not discussed in detail, but participants were recruited through diverse channels not solely restricted to clinical services, including a magazine and local radio. They stated that all participants reported self-harm without suicidal intent, but it is unclear whether this was the inclusion criteria, or participants independently endorsed this. | Participants were interviewed either in their homes or at a mental health charity office, providing a means of accessibility. Relational ethics were considered, such as ensuring participants knew they could take a break and/or end the interview which may support participant comfort and inclusivity by ensuring a trauma-based approach. | Both researchers identify as survivor researchers, centring user voice. The characteristics of the sample were briefly outlined but had limited diversity: all were White British and mostly young (in their 20s), only one man. Other socio-demographic information relating to socioeconomic background, race and ethnicity, sexual orientation and gender were not discussed. |
| **8** | Edmondson et al (2018) | This was not discussed. | The recruitment strategy involved advertising in both services and community organisations. In recruiting from mental health services, there is a risk of gatekeeping which was not acknowledged. Further details relating to the specificity of the advertisements were not discussed, for instance, relating to accessibility and inclusivity. | The researchers used a creative methodology, photo-elicitation, to elucidate participant experience, and promote expression and communication. Within this, the researchers emphasised the participant ‘driving’ the interview. This may serve to maximise accessibility and engagement. | The researchers involved service-users in study design, but how this was done was not described explicitly, reducing ability to conclude how far user voice was centred. The sample included two men, with variable ages. Other socio-demographic information relating to socioeconomic background, race and ethnicity, sexual orientation and gender were not discussed. |
| **9** | Gosling et al., (2023) | The researchers acknowledged the paucity of research examining the experiences of non-binary people, exploring the impact of individual and systemic discrimination. They further explored the interplay between self-harm and non-binary identities. | The recruitment strategy involved advertising in variable avenues, including a support organisation and social media. Further, the researchers attempted to target organisations focused on the intersect between LGBTQIA+ and people of minoritised racial backgrounds, however this was unsuccessful and only briefly mentioned. | The topic guide was informed by user involvement: it included easing participants into conversation and considerations were made as to participant safety and distress, including their ability to take breaks as required, which may support participant comfort and inclusivity by ensuring a trauma-based approach. | The researchers used an advisory board to contribute to study design, such as topic guides, supporting the centring of user voice. Although all participants identified as LGBTQIA+, an under-served group in self-harm research, the diversity of the sample was limited as to other socio-demographic characteristics. For instance, most participants were educated to a degree level and the majority were White British, with one identifying as Arab. However, overall, this research contributes to a greater understanding of self-harm experiences in non-binary peoples which has thus far been negated. |
| **10** | Gunnarsson (2023) | Not clearly discussed, however explores dominant ideas of self-injury in women, identifying clinical biases and focuses on underserved voices (middle aged and older women) | Recruitment not discussed | Explores how AE can allow the author to embrace subjectivity, reflecting on own experiences alongside included participants. | LE-led, author has explored LE of self-harm as someone who is older, explores social context, including relational interactions more than individual deficiency |
| **11** | Jackman et al., (2018) | This was not discussed. However, they explore self-harm experiences in an under-served community, transmasculine and non-binary youth. | The study was advertised widely across multiple, varied avenues, such as . No other information has been provided. | The researchers met with participants prior to interviews, as per their preferences (either phone or email). Interviews were conducted at a university only. | The researchers had a community advisory board contributing to all aspects of the research. However, detail was not provided so it was unclear how this was achieved. The sample was described as diverse in race/ethnicity, but further detail was not given. Most of the participants were students, limiting diversity. |
| **10** | Lockwood et al., (2021) | This was not discussed. | Participants were recruited from a previous online survey conducted by the researchers, but detail about that recruitment was not provided. | The researchers employed a card task to support conversations. These cards were pre-determined from self-report measures. Although this may promote accessibility by supporting participants to explore their experiences in interviews, it may also have constricted participant responses to be based around clinically determined ideas of emotionality in self-harm, which people with lived experience may or may not concur with. | The researchers used an advisory board to contribute to study design. The sample reported had limited diversity: 1aged between 16 to 22, 10 were white, 14 female; the remaining 5 identified as having mixed ethnic heritage, 1 was male. Other socio-demographic information relating to socioeconomic status, sexual identity and gender were not discussed. |
| **11** | Lundwall et al., (2025) | Not clearly discussed, but focusing on underserved group s | Recruitment was linked to a larger study | This was not discussed, exploration of questions asked in interview, interviewers trained in interviewing techniques but not explored in detail. | Focusing on how social distress affects autistic women, an underserved group, including two ‘insider’ autistic women to review developed themes (determined by researchers, not discussed about limits of this but section at end of results includes exploration of the insider researchers’ perspectives). Majority white identifying however, with no exploration of how this could be mitigated further considering that racialised communities are underserved. |
| **12** | Miller et al., (2021) | This was not discussed but overall, the study focuses solely on female adolescent self-harm, which is already frequently centred in self-harm research. | The researchers recruited from a clinical service only: they did so to ensure that participants could be reviewed by clinician in case of disclosure of self-harm, as the researcher was not a clinician. This potentially limits the fairness of recruitment by restricting the channels of recruitment to one type only. Details of recruitment strategy were not discussed, but suicidal risk did not affect inclusion. | A risk assessment was outlined, with the researcher assessing risk and taking clinical action taken as necessary. There was no mention of collaboration with the participant themselves in case of distress/risk. However, the researcher did ensure the participant was aware that they could stop the interview or refuse to answer certain questions. This could support comfort and inclusivity. | The researchers utilised a PPI group, reviewing the study design, questions and protocol, but did not specify details about how this was achieved, limiting ability to comment on whether this allowed for centring of user voice. The sample had limited diversity being all female, adolescent and solely from clinical services. Other details relating to race, ethnicity and other socio-demographic characteristics were not discussed. |
| **13** | Morris et al., (2015) | This was not discussed. | The researchers recruited from a personality disorder service, and specified clinical service use, cutting as the main self-harm methodology used by participants, as their inclusion criteria, thus restricting the diversity of the sample to a specific perspective. Specifics of recruitment, such as advertising, was not discussed. | Details relating to interviews, such as the employment of relational ethics and other considerations as to patient comfort and self-expression, were not discussed. | The researchers employed a service-user involvement group who commented on all aspects of the study: specific details were not given as to how this was achieved and what PPI members influenced, limiting ability to comment on whether this allowed for centring of user voice. The researchers  sample had limited diversity, being all participants were White British and mostly female, except one man. Other socio-demographic information relating to socioeconomic background, race and ethnicity, sexual orientation and gender were not discussed. |
| **14** | Mughal et al., (2023) | This was not discussed. | The researchers employed an inclusive approach to self-harm research, as supported by the PPI group, where all self-harm methods were included, irrespective of suicidal intent. The study was advertised openly and through diverse channels, including social media, community recruitment like university, colleges, libraries and self-harm third-sector organisations. | Participants were given an option for either face-to-face or telephone interview, all participants received a ‘staying safe’ sheet which listed support services. Participants were invited to first discuss how long they had been self-harming, which the researchers framed as supporting rapport building. Other aspects of the interviews were not discussed. | The researchers attempted to implement PPI through an advisory panel, but this consisted of people with self-harm, the public and clinical support workers, which fails to consider the power differential between healthcare professionals and people with LE. The wording of Tweets was refined by the PPI group. |
| **15** | Naz et al., (2021) | This was not discussed. However, the researchers acknowledged that rates of self-harm are particular high in South Asia in comparison to the global average, but self-harm and suicide data is lacking from the Global South. | Participants were recruited from a large public hospital only. Details of the recruitment strategy were not discussed. Participants with a mental disorder diagnosis were excluded. This limits the diversity of the sample – also has ramifications for validity, as many people who self-harm will have a mental health diagnosis. Unclear why people with a diagnosis were excluded. | The topic guide was said to be produced by a panel of psychiatrists and psychologists, meaning that key areas of discussion arose from a clinical perspective as opposed to user voice, potentially limiting inclusivity. A risk protocol and distress policy were put in place to offer support and counselling to participants. | The education, gender, age and self-harm method of participants were all reported. Race, ethnicity and sexuality were not reported, but it was assumed that all were Pakistani as the study took place in Pakistan. Thus, sexuality reports were complicated by its being illegal in the country of the research. Self-harm research examining the perspectives of Pakistani people is normally limited. |
| **16** | Norman et al., (2022) | This was not discussed. | Participants were not recruited from diverse channels, as they were drawn from a previous online survey about self-harm, that originally were recruited from Middlesex University and the public. Details regarding the specific advertisements were not given. | Steps were taken by the researchers to ensure participant wellbeing, including the use of a Visual Analogue Scale (VAS) at the start and end of the interview to gauge impact on mood. Participants were briefed about the voluntary nature of the research. | The researchers employed PPI; specific details not discussed in manuscript. The population sample was all women and young, aged between 18 and 29. |
| **17** | Norman et al., (2023) | This was not discussed. | Participants were not recruited from diverse channels, as they were drawn from a previous online survey about self-harm, that originally were recruited from Middlesex University and the public, but specificity not given. Details regarding the specific advertisements were not given. | Steps were taken by the researchers to ensure participant wellbeing, including the use of a Visual Analogue Scale (VAS) at the start and end of the interview to gauge impact on mood. Participants were briefed about the voluntary nature of the research. | The researchers employed PPI; specific details not discussed in manuscript. The population sample was all women and young, aged between 18 and 29. |
| **18** | Pollock et al., (2021) | This was not clear. | The authors excluded people currently in receipt of treatment, it is unclear as to why. Participants were recruited only from social work services, limiting the diversity and inclusivity of the participant pool. | This was not discussed. | The authors implemented an advisory panel consisting of one person with LE and then professional workers, failing to consider the power differential potentially existing between clinician/service-user. |
| **19** | Rebbettes & Bacon (2025) | Some gendered interpretations apparent in introduction but focusing on an underrepresented group, not discussed re EDI, but utilises a neurodiversity paradigm to be strengths-based rather than focus on individualistic deficiency | Recruitment restricted to social media, no information given in relation to sociodemographic information. | Discusses how efforts were made to accommodate communication needs, but detail not given | LE-led, acknowledges that the lack of inclusion of autistic peers is a limitation. |
| **20** | Rosenrot & Lewis (2025) | Explored the implication of stigma on limiting people’s disclosure of self-harm, potentially amplifying feelings of shame. Introduces self-harm as particularly apparent among youth and young adults, contributing to the idea that adults above 25 do not engage in self-harm. | Recruitment was from a larger study, potentially limiting the ability to diversity recruitment. Indeed, sample was predominantly female. Race and ethnicity not discussed, nor highlighted as a needed area of research due to exclusion of people of colour from research. | This is not clear, some discussion around interviews being conducted in a private lab space but other aspects, e.g. building rapport, not discussed. Debriefing sheet given at the end, limits to that approach, as pushes forward the idea of the individual being ultimately responsible for their own distress. | Co-led with someone with LE, but no other inclusion with peers with LE, limiting inclusivity. |
| **21** | Russell et al., (2010) | This is not explicitly discussed but the authors explore variable interdisciplinary constructions of self-harm, this creates a diverse and inclusive picture of the phenomenon. | The authors recruited from across the country. Mental health clinicians were contacted to ask them to approach clients who engaged in self-harm. This yielded a very small sample of men. The authors did not consider the risk of gatekeeping in only contacting clinicians. Further, the implication was that only people under a mental health service were recruited, limiting diversity. | The authors detailed the various suggestions from the ethics board and people with LE via local MIND group to achieve a safe space for people. The authors then explored what the interview entailed and what this conversation involved. For example, the author explored having an “engaged curiosity” in discussing LE with participants. The authors did discussed the participants’ experience of taking part, as well as arising thoughts and feelings from the participant and the researcher conducting the interview. The researcher provided participants with the choice to see a copy of the research. | The authors consulted with a local MIND group for people who self-harm to ask for their guidance around research process. It is not made clear when this conversation was conducted however – e.g. at the very beginning or once research priority had been decided. |
| **22** | Sabo et al., (2025) | Not clearly discussed | Issues with gatekeeping not explored, issue with fluency in English, focuses on recruitment from mental health services, plays into the ‘clinical risk’ of self-harm, negating how majority of people do not have medical contact etc., | Participants offered various ways in which accessibility cold be promoted, e.g. written responses to interview questions | Focusing on underserved population in understanding self-harm, autistic-led, no PPI, some reflexivity, examples of what was reflected on were not given |
| **23** | Stänicke (2021) | This was not discussed. The authors solely consider the perspective of young women in a clinical population, briefly discussing the variation of self-harm across genders. | Participants were not recruited from diverse channels and focused on a population of young women from a clinical sample (outpatient clinic), which reflects current contemporary self-harm research. They utilised theoretical sampling to establish ‘sample-heterogeneity’ relating to age, gender, frequency and form of self-harm, cultural and socio-economic background, and education. Other details regarding recruitment were not given. | The topic guide was developed with the advisory panel; the researcher confirmed her understanding throughout to support the participant to explore their experience openly. During the interviews and in feedback meetings, the participants were given the opportunity to add information, and the PPI group commented on interview guide and manuscript. | Three people with lived experience of self-harm were involved at all stages of the research process. Some data was given regarding the socioeconomic background of the participants, which was variable, but not about race and ethnicity, making it hard to comment on whether the population was diverse or not. |
| **24** | Troya et al., (2019) | This was not discussed. However, they examine an under-served community, older adults, exploring how they are often negated. | The study used extensive and diverse channels of recruitment, including public avenues, social media and third sector groups. | The researcher ensured that the time and venue of the interview suited the preferences of the participants. | They used PPI in their study and explored this in detail in a separate reflective piece. Specificities relating to socio-demographic, such as race and ethnicity was not offered, but information related to age, gender and health status was given. |
| **25** | Williams et al., (2023) | This was not discussed, focusing on an underrepresented population (LGBTQ+) which is positive, discusses minority stress, issue as per Kendi’s exploration of ‘stress’ depicting abuse as a passing inconvenience as discussed in main body of manuscript. | Various recruitment strategies used, with participants holding diverse identities, not exploring racialised communities. | Reflexivity discussed in detail in relation to interview procedure, i.e. participant/researcher. Discussion in interview of encouraging participants to discuss self-harm as per their experiences. | Inclusion of advisory group of gender diverse and sexual minority youth with experience of self-harm, and LE-led. |
| **26** | Witcher et al., (2025) | I would say issues of exclusion regarding strictly defining self-harm and excluding various methods which, variably, many people with LE may describe as also being self-harm (e.g. eating distress and substance use). Issues with naming self-harm as an addiction because not acknowledging how discourses ‘leak out’ and addiction may be used as a means of justifying or rationalising engagement in the behaviour, but ultimately fails to consider how addiction is shaped within social circumstance etc., they also define self-harm as a behaviour, with a brief consideration of it as an expression of distress, but then discuss it diagnostically. They do acknowledge that community samples involve people who self-harm. They use a vignette methodology with the intention of improving accessibility in exploring sensitive topics. | Variable avenues for recruitment, attempts made to recruit men. | They discuss use of a pilot interview, with one person with LE, who shared that they felt comfortable disagreeing with the researcher, positive of course but leads to the issues of relying on one voice with which to speak to all people, so does not necessarily confirm that participants could have disagreed with the vignettes (i.e. that self-harm is not addictive). | LE-led with input from those with LE. Attempts made to recruit men as initially recruited 7 female participants. Only recruited one man. No other underserved identities noted. Member-checking to promote methodological integrity. |
| **27** | Woodley et al., (2021) | This was not discussed – does explore multiple different theorisations of self-harm which is positive | The study used only one avenue for research recruitment, and there was no detail given as to how this was undertaken. | This was not clear. | One of the co-authors was a service-user researcher. Information was given regarding gender and ages, but not sociodemographic information. |

**Supplementary Material 4:** Coding examples

| ***No*** | **Author(s)** | **Example quotation(s)** | **Accompanying explanation by author(s)** | **[Author 1]’s interpretation, as contextualised within research objectives and LE of self-harm and professional experience as a nurse** | **Associated code/theme** |
| --- | --- | --- | --- | --- | --- |
| 1 | Jackman et al., (2018) | “And kind of within myself realised that, like I don’t particularly feel shame or guilt or anything [about NSSI]. Like I found a thing that helps me and, you know, ideally, you know, someday there would be a planet where I don’t have to do that but as of, like the situation that I’m in now this is the best way for me to survive…” (p9-10). | “In an environment with little support and few resources, participants described NSSI as a simple and effective way to deal with stress or aversive emotional states” (p9). | - (RO1, RO2, RO3): In referring to aversive emotional states prior to providing the quotation, the authors are potentially drawing upon the affect regulation perspective of self-harm, linking the phenomenon to people with LE ‘not being able’ to access adaptive resources to cope with an environment with limited social support. This centres the person. - (RO3, RO4): An alternative interpretation is that wider sociocultural normativity of binary genders create distress for people with transgender and non-binary identities, resulting in self-harm to cope with a world that does not accept their ‘non-normativity identity’, directing the negativity implied from ‘outer’ experiences onto their bodies (‘inner’). | 1) *Dynamic flow* under *Liminality of Self-harm;* 2) *Social difference, discrimination and victimisation* under *Social and relational experiences;* 3) *Linking self-harm to intersecting identities and social experiences* under *Web of self-harm* |
| 2 | Lockwood et al., (2020) | “I’m trying to be like, be an adult, and I’m like, I feel like, I have little tolerance when I’m acting out because I’m like – You’re not 16 anymore, like stop!” (p15). | “Some described a changed emphasis in conscious and deliberative effort over time suggesting that they were now better able to take a step back from the emotional reactivity and recruit effective techniques of internal management” (‘Laura’, p15). | - (RO1, RO2): The authors note that people with LE may develop more ‘effective techniques of internal management’. This aligns with clinical conceptualisations of good/bad, ineffective/effective coping strategies, whereby self-harm may be considered a ‘maladaptive’ means of coping with ‘internal experiences’, reinforcing ideals of individualised emotionality. - (RO2, RO3, RO4): ‘Laura’ here potentially describes the influence of wider sociocultural conceptions of self-harm as a ‘childlike’ behaviour (as discussed by Gunnarsson, 2024, included in this work, as ‘reverse ageism’), highlighting how the perceptions of people with LE may be influenced by an awareness that other people/society disapprove of self-harm or apply a negative lens on the phenomenon. This has potential important implications for people’s engagement in self-harm ongoingly. | 1) *Divided binary* under *Liminality of harm;* 2) *Bad and unwanted* under *Experience of the harm.* |

**Supplementary Material 5:** Initial Codebook

| **Overarching Code (Preliminary theme)** | **Code** | **Comment** | **Quotation** |
| --- | --- | --- | --- |
| Theme 1 | Labelling and diagnosis | Participants discuss the value or drawbacks of being diagnostically labelled. Some people with lived experience note that it means clinicians may put you in a box. | “I always say, I know people call it when you get diagnosed being labelled, I actually liked that, I wanted to be diagnosed because it gave me an answer, like it wasn’t just oh I’m feeling because oh I don’t know, it’s like I’m feeling this way because I have this or have that, like there’s a reason for it.” |
|  | Medicalised understanding of self-harm | Participants occasionally understood and described their self-harm within biomedical frameworks, such as ‘pain-relieving chemicals’. | “I think the first time it was associated with a kind of rush and a buzz, it was more of a sort of, the blood, and the sort of you know adrenaline.” |
|  | Awareness and impact of others’ perspective of harm | Participants often were aware of the implicit or explicit opinions of other people about self-harm, and this influenced self-understanding, concealment and disclosure and continuing engagement. | “In most cases, participants suggested that they did not ‘mind’ their scars, but simultaneously indicated concern and anxiety around what others might think – or assume – on seeing scars” |
|  | ‘Curing’ the harm | Participants discussed the removal of harm, and by association scars, to ensure a return to a ‘pre-injury’ state, in line with medical (and societal) conceptualisations of reducing symptoms to achieve a cure. | “Well I think I started self-harming when I was 17 in high school and it got it was real bad for about two years and I would do the whole you know we’re gonna we’re gonna stop doing this because it’s bad and my best friend hates that I do it, and then keep doing it”; “You say I should stop, I shouldn’t do this to myself. You say you’ve had enough. You can’t help if I don’t want the help. Do you not see I just don’t need it. Really, my heart and my brain is what’s bleeding, these these these are just cuts” |
|  | Putting onus on the person to communicate experience | Participants with lived experience felt there was a separation between them and ‘mental health people’ due to their ‘inability’ to communicate their experience ‘effectively’. | “I can’t describe it and they can’t see the rain” |
| Theme 2 | Justifying the harm | Participants appeared to attempt to justify or defend the harm by positioning it within understandable frameworks such as emotional regulation, biomedical theory or considering it as a necessary tool. | “By no means do I promote or endorse self-injury as a way to cope or express emotional pain. It is what I had at the time, and what countless others have at their immediate (and often desperate) disposal.” |
|  | Harm is positive | Many participants identified self-harm as serving a positive, helpful or needed purpose that helped them at a difficult time. People may affirm its use. | “Because that one was so bad, it almost serves as a sign, I don’t need to cut, I’ve got that, it’s like a badge, I think if I hadn’t done that, my arm would have been a lot more – covered in small cuts.” |
|  | Harm is bad | Some participants identified self-harm as ‘unhealthy’, ‘bad’, ‘negative’. | “We’re gonna stop doing this because it’s bad” |
|  | Harm is rational | Linked with the above code ‘justifying the harm’, participants attempted to rationalise self-harm by positioning it as a rational or logical response to pain. This appears to defend its use. | “For me, cutting was a rational response during an irrational period of my life” |
|  | Ambivalence and paradoxical dissonance | Participants often demonstrated conflicts in how they viewed self-harm, both framing it as bad/positive, good/bad, destroying/saving (as above codes). Further, participants might experience a push-and-pull between feeling not wanting to self-harm, but wanting to continue to do so. They may also feel sometimes comfortable with scars but sometimes seek to cover them. This seems often tied with others’ perceptions. | “I suppose there’s a bit of a disparity cos, in my mind I sort of feel like I’m OK with it, like I’m perfectly happy with what I’ve, you know I don’t have any reg-I don’t really regret doing it or I’m really ashamed of it, or anything like that, but at the same time I’m not, I don’t, wouldn’t want to just openly talk about it at work (…) I think that’s basically cos of, I think they might have misconceptions”; “as much as I was ostensibly destroying myself, cutting served to save my life” |
|  | Habitual response | Self-harm becomes a habitual response to a trigger. | “In what had become a habitual response to unbearable emotional pain, I reached for a razor blade – one of many I kept on hand” |
|  | ‘To look beyond the behaviour’ | Participants discussed the importance of considering what the underlying experience was for the person with self-harm, rather than simply considering the behaviour as the main component. | “These are just cuts” |
| Theme 3 | Experience of emotionality, feelings and sensations (in relation to being oneself) | The underlying phenomenology of being a subjective individual and the language used to refer to and explore one’s emotional inner world. | “The situation seems to ‘spiral’ and I’m whooo losing it”; “The image played over and over again. With it came a barrage of messages: ‘you’re worthless’, ‘you’re nothing’, ‘you’re weak and pathetic’”. |
|  | Self-identified difficult experience or situation | Participants described difficult situations in their life narrative, such as implicitly referring to social conflict or perhaps previous abuse. | “I was haunted by painful memories.” |
|  | Dualism and division of the self | Head/body, self/other, physical/emotional, inner/outer, external/internal | “Not understanding things (…) and trying to make something of, whatever’s in my head, to make it into something understandable and manageable” |
|  | Strong but broken | Some participants wanted appear strong, or hardy, but felt that really they were broken. | “Stay upright, stay together and not cross those boundaries so people would find out what was going on because that was something that I couldn’t do so I had to internalise it.” |
|  | Being set apart from others, hiding inside oneself | Many participants felt cut off from others, but also might not inform others of their distress. Self-harm was a means of ensuring this as a way of protecting the self. | “I was an undergraduate student in psychology and seemingly had it all together; at least that’s what others thought. Like an iceberg, there was plenty below my surface.” |
|  | Limits to language – ‘Having no words’ | Many participants may describe self-harm and their experiences through metaphors or otherwise argue that certain situations are difficult to verbalise in words. | “Well I don’t know, um I don’t know if I ever know what I want to say. I know for sure what I’m feeling, and how I’m feeling inside and how I want to feel but I don’t know how to, I don’t know how to say it, like I don’t know how to find the words to use. And so I just don’t try.” |
| Theme 4 | Experience of emotionality, feelings and sensations (in relation to arising harm) | The phenomenology of enacting harm against the self and the associated inner psychic experience as motivating engagement. | “That’s just how I feel I guess, there’s so many thoughts and ideas and feelings and everything just sort of, I don’t know, it’s just everywhere” |
|  | ‘Inner turmoil’ and chaos | There is ‘too much’ ‘inside’, so self-harm reduces ‘the storm’. | “It was the only way I knew to quell the storm brewing inside.” |
|  | Bad for being oneself – ‘evil inside’ | People with lived experience see themselves as irrevocably bad, evil, hateful or disgusting. | “I just have sort of warped body image, I don’t know if that’s part of the self-harm, I hate this body, I can’t look in the mirror” |
|  | Control but out of control | Participants discussed concepts of ‘control’ within harm experience, but on the other side spoke about feeling out of control. | “Right, regain control, this is what I’m gonna do.” |
|  | ‘Cut a line in the sand’ | Participants tended to describe a before/after with self-harm, where the before was messy and the after was calm. | “Self-harm is like a full stop, like punctuation, it’s punctuation, it’s a sort of punctuation to moods or emotions or to a series of memories.” |
|  | Release or relief from something, come out | Many participants describe self-harm as releasing ‘something’, and by association leading to a sense of relief. | “… it was literally like I could feel it and hear it sort of like tearing open … that was it, that was the one, it was like, it’s worked this time (…) and it’s like releasing something” |
|  | Stigma, guilt and shame | Many participants described feeling shame and guilt for enacting harm, but also feeling what they felt to be ‘inappropriate’ emotionality. | “At the same time, cutting silenced me: it engendered a profound sense of shame for inflicting such harm on my own body” |
|  | Psychic experience motivating, urging harm | Describing thoughts and associated images that come up in relation to harm, either urging engagement or linked with harm. | “My thoughts were marred by self-abhorrent messages, urges to harm myself and a belief that I was destined to feel the way I did.” |
|  | Chosen practice of self-harm | Particular methods of self-harm were described, with one mention of a particular self-harm episode. | “When I spoke with her, Anna reported cutting herself regularly and increasingly extensively.” |
|  | Self-harm gave me my voice | Harm substitutes speaking about one’s experience or helps a person express that which they cannot express. | “I don’t use my words, so the pressure builds, then I cut and that’s how I deal with that” |
|  | Speaking with and exploring the body | Harm is distress written upon the body. | “It is about adornment and celebration (…) and in a way my scars as well, actually, cos I do think they’re really beautiful and they’re part of my experience, my history.” |
|  | Transformation of the self | Harm is an attempt to transform or improve the self, and in the long-term, can hep change the situation or person for the better. | “Ultimately, what I learned through my experience makes the person I am today – a person I accept without shame.” |
|  | ‘Self-harm proves it real’ | Harm aims to achieve the following: a) Transformation into something visible and tangile, emotional pain into physical pain; b) peeling back layers to see inside the self, to understand oneself | “I just want it to sort of stop and slow so I can sort it, into something that I can, understand and deal with, and erm, yeah I guess sometimes when I can’t do that and I just can’t do it and it’s just really frustrating, I need to feel something, that I know is real and it’s there and it’s concrete, my arm hurts, it’s bleeding, it’s a feeling, instead of just confusion and not understanding things” |
|  | ‘It’s worked this time’ | There may be a ‘rightness’ with self-harm, so that a person needs to enact harm until it achieves this sense. | “That was it, that was the one, it was like, it’s worked this time that” |
|  | Suicidality | Harm was sometimes intertwined with feeling a need to end one’s life. | “Not surprisingly, my situation worsened. Despite the momentary relief cutting provided, the more I cut, the more depressed I became and, in turn, the more hopeless I was about recovery. It was a never-ending cycle. Before I knew it, I was suicidal and even took actions to end my life.” |
|  | Progression | One’s engagement with harm may change and progress over time, so that its function and reasons motivating engagement may alter. | “Anna’s account implied less control over the progression of self-injury, and the generation of ‘bigger’, ‘deeper’ scars: scars which were less amenable to attempts to reduce their appearance.” |
| Theme 5 | Concealing and minimising scars | Participants felt conscious of scars, and a need to hide them from others. | “I also looked into like you know, trying to see, er, ways of kind of you know, making scar, tissue look less, obvious and stuff erm, … I got this quite interesting stuff that was like em, … kind of like em, a gel pad, a silicone gel pad […] that kind of, comp [ressed] and actually, made- you know you had to wear it, like every night […] and then, like it consistently kind of pushed it down […] but then if you don’t keep using it you know it sort of, they sort of show more […] and you end up kinda going back to the, state […] but, em, that ﬂattened it off […]so that, you know that was again, kind of, you know trying to kind of, get to the point where you don’t feel kind of worried about kind of”. |
|  | Corporeal aftermath of the harm – wounds and scars | Participants spoke about the harm after an episode, in the form of wounds and scars. | “Today my body is covered in scars; they represent a narrative of past despair that I carry with me everywhere I go.” |
|  | Learning to speak | Participants learned how to speak about their harm and experiences in ways that were right for them. | “I guess like I struggle quite a lot to put my feelings into words and music’s almost a replacement for that, like I can listen to a song and be like exactly, damn it, that’s bang on, that’s exactly how I feel.” |

**Supplementary Material 6:** Finalised codebook

| **Theme** | **Sub-theme** | **Code** | **Quotation Examples** |
| --- | --- | --- | --- |
| *Interpretative Theme, as shown in Discussion:* ***Liminality of self-harm*** | ***Divided binary***  As apparent in psychiatric discourse, many researchers and some people with lived experience appeared to adhere to a dualistic understanding of self-harm, such that the phenomenon appeared subject to static, divided categories. For instance, good/bad, head/body, inner/outer, choice/lack of choice, personal/social, destruction/care, repression/expression, vulnerability/strength.  ***Dynamic flow***  Some people with lived experience and lay researchers sought to explore wider sociocultural influence, framing self-harm is being more free-flowing, and contingent not only on an individual by themselves, but as existing within social contexts. | | “I locate my experiences of self-harm as an experience in which I was exercising control over myself, my body, my life, but also as something that happened to me, which at times felt frightening or beyond my ability to even comprehend” (Heney, 2020)  “…you’ve got to choose this or that, there’s nothing in between (…) no space for confusion” (Simopoulou & Chandler, 2020) |
| **Theme One: What is self-harm?** | Most researchers broadly considered self-harm as first and foremost a self-destructive behaviour, with some research specifically stating common methods, such as cutting, burning, and self-poisoning. Other means of self-harm, such as bruising, was less commonly identified as objectively self-harm. Often, self-harm was perceived as a coping mechanism, skill or strategy, commonly employed to manage or regulate affect and/or emotional state. In some other research, self-harm was also contextualised as a means of self-punishment, communication and expression and self-protection. Self-harm was commonly constructed as indicative of psychopathology and mental illness. Occasionally, self-harm was linked to traumatic events and attachment difficulties. People with self-harm are often considered in negative terms, such as the common ‘attention-seeking’, and by extension, self-harm is thought to be both morally bad and wrong. This means it should be contained and stopped. | | “Explanations for self-injury in particular have argued that it is a method of ‘coping with’ unbearable emotional pain by inflicting physical pain on the body” (Chandler, 2013)  “The medical community views ‘superficial’ self-harm as a nuisance at best” (Gurung, 2018) |
| **Theme Two: The web of self-harm**  For ease, this theme is divided into four sub-themes, but it is recognised that all appear to occur dynamically and simultaneously, not as dualistic entities. | **Sub-theme 1: *Living in a pressured world***  People with lived experience identify a variety of traumatic and difficult experience in both childhood and throughout their life, including abusive circumstance, family discord and bullying, with associated lack of support and care. | Bullying, disclosure of abuse, family discord, lack of support and care, loss, mental distress, negative experiences in mental health services, overwhelming social situations, physical/verbal/sexual and emotional abuse, relational conflict/resolution, risk-taking, school, social difference, discrimination and victimisation, sociocultural obligations (‘Faultless individual’), suicidality | “Coming from like the depression, anxiety, uh the self-harming. I had an eating disorder, like, not only was it the abuse coming from my father and what he told me, what how he tried to mould me into the perfect girl, under his eyes, if you want” (Female participant, 16 years, Collin-Vezina et al., 2021).  “So, it wasn’t just problems with my dad, it was problems with my grandad, some sexual abuse. And uh with my dad it was a lot of physical and emotional abuse. I don’t know, there might be other things that went on as well, but I don’t know, I find those hard to look at” (Female participant, 65 years, Troya et al., 2019)  “My extended family is the root of a lot of my mental health issues.” (Non-binary person, age not given, Gosling, Pratt & Lea, 2023) |
|  | **Sub-theme 2: *Embodied psychic experience as oneself* changed to *Knowing oneself* changed to *Embodied Sensations***  People with lived experience describe multiple, often all-encompassing emotions and feelings that they feel unable to contain. Most commonly, this was seen as internal turmoil or tension, that felt incommunicable to themselves or others, either because they could not find the words or did not think the words would be understood. Others described a diffuse sense of badness, self-hatred, disgust, self-loathing, feeling dirty and worthlessness. This was discussed even in the absence of self-harm, but many then linked it towards the practice. Some identified their arising emotional experiences of anger, mental distress, powerlessness, and self-blame, as well as their responses to it, as grounded within adverse childhood experiences, trauma or because of personal intersecting identities, such as LGBTQIA+. Describes ‘inner’ psychic experience, such as emotions, feelings and sensations that precede and mediate the physical act, occurring dynamically together. People with self-harm may experience a building pressure of chaos and turmoil that feels incommunicable, alien and incomprehensible. Others explore a sense of unacceptable ‘badness’ that is experienced within; arising anger and negatively is then explored by directing to the self through harm. However, some explicitly understand this as linked to traumatic experience in the past and present that is held within the body. Overall, many people with lived experience identify self-harm as a means of working on and with emotions, engaging with but not avoiding their ‘inner’ discomfort and chaos. | Alien and incomprehensible, cognitive responses to emotionality, disconnection to one’s body and/or oneself, feeling different and out of place, feeling unable to assuage, contain or negotiate emotion, incommunicable and difficulty communicating, intersecting identities, lack of coherent self, psychic experience intertwined with relational and social conflict, strong but broken (‘hardy persona’), turmoil and tension, unacceptable badness (‘the evil inside’), Ideation, suicidality, the catalyst (a build-up of chaos) | “I was like feeling so bad and I couldn’t … understand what was going on inside me, I was like hurting so much (…) I couldn’t understand it” (Female participant, age not given, Chandler, 2013)  “One ‘cutter’ believed that simply describing her childhood trauma out loud would cause physical harm to her therapist. Eventually, she threw a packet of razor blades at the psychologist, telling him that the blades could express what she could not.” (Gurung, 2018).  “You had to (…) lie across your bed on your stomach with your arms at your side and she would be hitting you with a cane over and over (…) you just had to take t and take it (…) you had no choice but to put your hands in the way because it was just too much and you would just get even more then because you’d shown some sort of emotion really, you weren’t even allowed to cry” (Morris et al., 2013).  “Sometimes I start to panic about things, and the only way I can stop panicking about it and think rationally it is (…) cut myself” (Female participant, 33 years, Chandler, 2012)  “The build-up of emotional turmoil and tension” (Donskoy & Stevens, 2013)  “Five participants spoke about a build-up of difficulties in multiple aspects of their lives, leading to a ‘breaking point’ which then led to self-harm” (Gosling et al., 2023)  “What you’ve got really is a huge accumulation of experiences both at home and at school and at work that had sort of built up and really sort of hitting against the dam wall, and then finding a breaking point” (Gosling et al., 2023) |
|  | **Sub-theme 3: *Enacting an embodied practice***  Describes the corporeal component of self-harm as the wounding enacted on the outside of the body. People with lived experience understand their self-harm as beginning mostly in early to mid-adolescence, and as enacting as a physical practice of wounding the outside of their body, but that transfers emotional pain into physical pain to legitimise and make their experience tangible and visible. People with lived experience describe planning episodes of self-harm as well as engaging quickly and immediately to soothe turmoil. Some people find the release of blood symbolically important to the practice and find comfort and meaning with the scars and wounds left behind. Likewise, people with lived experience recognise self-harm as soothing, providing a dampening to the chaos. Despite people emphasising the physical practice as important, they recognise that there is a need to look beyond the behaviour when supporting people with lived experience. | Awareness and impact of others’ perspective of self and harm; ‘a full stop’, anger and negativity unto self, being an outsider, set apart and hidden from others, creating more shame (cyclical nature of harm), cultivating and eliciting, directing negativity to self instead of others, linking self-harm to social contexts, seeking to connect to others, temporality | “The idea that distress is contained in the body, until it becomes too much and is ‘released’ in ‘violent activity’” (Chandler & Simopoulou, 2021)  “Participants’ descriptions of the ‘work’ that they did during self-injury” (Chandler, 2012)  “Francis suggested that his self-injury had partly been oriented towards eliciting emotions where previously he felt ‘numb’” (Chandler, 2012)  “Just feeling something” (Lockwood et al., 2021)  “Sometimes they just needed to see this blood to prove that they are still alive” (Miller et al., 2021)  “Cause I know for me not being able to express anger especially was a massive thing that led me to turn that in on myself, and not having an outlet, where it was kind of like you are a woman…” (Chander & Simopoulou, 2021) |
|  | **Sub-theme 4: *People’s relationships to self-harm***  People with LE describe complex relationships to the self-harm where they both found it helpful to rely upon but also thought it was not normal and harmful. People with LE were clearly conscious and aware of wider social constructs of self-harm as ‘bad’, and anticipated negative reactions from others, as well as directly experiencing discrimination. The impact of this was that people sought to conceal self-harm further, lying about it and changing where they self-harmed on their body, people with LE also felt at fault, and this could lead to more self-harming. Thus, social ideas fed into individual perceptions. | Amplified badness, alienation and shame, more self-harming, hiding oneself further, justifying the harm, self-harm is harmful, bad and unwanted, harm is positive, harm is rational, harm is something to rely upon, self-harm is shameful | “… I do think it is something that I know so intimately, and it knows me (…) I have a relationship with it that I don’t have with anyone that I know in person, and no-one will know that relationship either, which I think keeps it being such an intimate thing”  “It gives a perfect reason for why self-harm is the right thing to do, because if I’m a bad person then I deserve that pain and that sort of state of mind and everything that comes with it”  “t’s absolutely degrading and you feel crap every time you look at yourself, every time you see them scars” |

**Supplementary Material 7:** Data extraction of self-harm conceptualisations across literature

| **No** | **Author(s), Year** | **Self-harm terminology** | **Self-harm definition and/or conceptualisation** | **Self-harm methods reported** | **Main findings** |
| --- | --- | --- | --- | --- | --- |
| **1** | Rebbettes & Bacon (2025) | Self-harm | The deliberate act of self-injury or self-poisoning, regardless of motivation or suicidal intent. Draw upon intersectionality theory to argue that experiences of autistic women cannot be isolated from identity and sociocultural context. | Cutting, burning, genital self-mutilation, head banging, skin picking, self-embedding, alcohol and drug use | Feeling different and not fitting in, autistic burnout, overwhelm versus numbness as driven by external demands, considered self-harm and the persisting need to self-harm, discussions of receiving autistic diagnosis |
| **2** | Boyce (2021) | Self-harm | The deliberate act of self-injury or self-poisoning, regardless of motivation or suicidal intent. Explores the enduring assumption that self-harm is an adolescence behaviour, drawing upon survivor movement to note self-harm as a coping strategy across the lifespan for distress, other research cited examined how self-harm negotiates the private and public self. | Head-banging, other non-marking, cutting, biting, hitting, eating distress | The role self-harm has in assuaging strong feelings, methods of self-harm, changes to self-harm engagement over time, |
| **3** | Brown et al., (2022) | Self-injury, NSSI | Intentionally and directing injuring one’s own body tissue, without suicidal intent, used to self-punish, prevent suicide, with the most commonly endorsed function of NSSI is emotional regulation. | Cutting, burning, scratching and biting | Shame is experienced as a social and relational emotion, feelings of failure, being trapped, dangerous or contaminated and hidden or exposed. |
| **4** | Chandler (2012) | Self-injury, self-harm | Self-poisoning and self-injury, regardless of motivation or suicidal intent. However, they note definitional problems has compromised understanding. For instance, in relation to practical aspects of the behaviour. | Cutting, burning, hitting | Self-harm marks a means of ‘doing’ emotion work, highlighting the under-recognised importance of examining the practical, corporeal practices that can be involved in emotion work. Reflections include the sociological and theoretical significance of examining self-injury as embodied emotion work. |
| **5** | Chandler (2013) | Self-injury, self-harm | Self-injury is a form of self-harm, defined as self-injury or self-poisoning irrespective of the purpose of the act. However, as previously, they acknowledge the contentions around self-harm. | Cutting, burning, hitting | Exploring pain in self-injury, where self-harm alleviates or transforms emotional pain through the infliction of physical pain, whilst also exploring pain as irrelevant or non-existent, self-injury as ‘opposite’ of painful (pleasure), and pain as a central feature of the practice. |
| **6** | Chandler (2014) | Self-injury, self-harm | People who self-injure can be seen to occupy an uncertain position, one which unsettles notions of sanity and madness, dramatically breaching imagined boundaries between physical and mental health, no other definition offered. Self-harm noted as encompassing self-injury and self-poisoning. | Not discussed explicitly | Discussing with a body marked by self-injury, including trying to return to a pre-self-injured state, exploring how self-injury works as a response to chaos, providing insight into the transformative, positive nature of self-injury and permanent scarring. |
| **7** | Chandler & Simopoulou (2021) | Self-harm, refers to the variable terminology | Self-injury or self-poisoning, irrespective of the apparent purpose of the act. Acknowledges the constraints of this broad definition. | Not discussed explicitly | Making sense of the gendering of self-harm, focusing on a series of dualistic Cartesian ‘cuts’ between male and female, violence and vulnerability, and inside and outside. |
| **8** | Donskoy & Stevens (2013) | Self-wounding, self-harm, self-injury | Drawing upon the medical discourse, survivor research, socio-anthropology, with explicit definition but providing an overview of the complexity of self-harm | Not discussed explicitly | The narratives of self-wounding show that the first episode occurs in a complex landscape of interactions between events and emotions, self-harm described as associated with a strong need to gain or regain control of emotionally charged and chaotic environment. |
| **9** | Edmondson et al., (2018) | Self-harm | Focus on explanations for repeated self-harm as a reaction to disordered affect regulation or interpersonal relationship problems, highlighting that this has been criticised by service-users to problematised rather than understand | Not discussed explicitly | Self-harm was discussed as a response to distress, to achieve mastery, as protective and as a language or form of communication. |
| **10** | Gosling et al., (2023) | Self-harm | Causing physical harm to one’s own body, with or without the specific intent to die | Cutting, burning, scratching, hitting oneself, self-poisoning | Exploring how non-binary young adults describe growing up outside of the binary, family discord and the pain of living in a cisnormative society, as associated with self-harm. |
| **11** | Gurung (2018) | Self-harm, also refers to how self-harm is referred as self-mutilation, self-injury, cutting, parasuicide, NSSI and deliberate self-harm | Explores how self-harm is presented in academic literature and popular culture as a pathological act of ‘intentional injury to the outside of the body, mainly through cutting, but including scratching, burning, biting or hitting. | Discusses different types of self-harm methods as medically categorised according to severity from cutting, scratching, burning, biting, hitting, eye enucleation, castration and limb amputation | Exploring the narrative of those who engage in such acts that self-harm may be better construed as a meaningful embodied emotional practice, bound up in social (mis)understandings of psychological pain and how best to attend to such pain, suggesting that self-harm practices are embodied, socially situated acts of healing, survival, and self-creation in a physical attempt to retell complex, fragmented stories of abuse, existential angst, trauma and loss of self. |
| **12** | Gunnarsson (2021) | Self-injury | Discusses how self-injury is often discussed within an objective perspective, but may have multiple meanings and function, as a mechanism to manage emotionality, identity, survival and control | Not explicitly discussed | Exploring how shame and self-injury reproduce each other in social interaction, through the role-taking process of seeing the self as one thinks others see oneself |
| **13** | Gunnarsson (2021) | Self-injury | Not explicitly discussed | Not explicitly discussed | Exploring shame and shame reactions in an intimate relationship, how shame was restored through the other’s validation and acceptance, or how it led to more shame managed by self-injury. |
| **14** | Gunnarsson (2022) | Self-injury | Discusses how self-injury is dominantly deemed as a symptom of mental health issues, as an individual psychopathology; it is considered a deviant practice and thus not socially sanctioned or culturally acceptable. | Cutting and burning | Reflecting on how self-inflicted scars can have the same meaning as self-injury to soothe the self and mange emotional pain, and how the marked female body can be resistance to the cultural idea of femininity. |
| **15** | Gunnarsson (2023) | Self-injury, refers to the use of other terms such as ‘self-mutilation’, ‘delicate self-cutting’, ‘non-suicidal self-injury’ and ‘self-harm’. | Explores how self-injury is considered a deviant and culturally unacceptable behaviour, that is individualised. This conceptualisation is fraught with a host of different conceptions, myths and expectations of the person who carries out this act. The author considered how self-injury is ultimately a social practice, that both produces and is a product of embodied social experiences that, I different ways, are positioned in time and space. | Cutting, burning, breaking bones, inserting needles under the skin or other acts that injure the body’s tissues in some visible way, often leaving permanent scars | Exploring how four women and the author with experiences of self-injury in adulthood, use, internalise and speak through dominant discourses of self-injury. This includes considering the stereotypical notion that self-harm is a ‘young person’s practice’, and the ageism bandied against adults with self-injury. |
| **16** | Gunnarsson (2024) | Self-destructive behaviours and thoughts | Self-destructive thoughts and behaviours, while harmful, can also serve as coping mechanisms and contribute to a sense of aliveness and self-cohesion. | Not explicitly discussed | Discussions of how self-destructive thoughts can persist, breaking up the monotony of daily life and provide a sense of identity and aliveness, exploring that despite living a fulfilling life, the temptation of past behaviours remains with me, highlighting the complex relationship between self-destruction, identity and societal expectations. |
| **17** | Gurung (2018) | Self-harm, notes how it is also referred to as self-mutiltation, self-injury, ‘delicate self-cutting’, ‘cutting’, parasuicide, NSSI, and DSH | Discusses how it is generally presented in academic literature and in popular culture as a pathological act of ‘intentional injury to the outside of the body, mainly through cutting, but including scratching, burning, biting or hitting’ | Cutting, scratching, burning, biting or hitting | Explores how self-harm practices are performing embodied, socially situated acts of healing, survival, and self-creation, in a physical attempt to retell complex, fragmented stories of abuse, existential angst, trauma and loss of self. |
| **18** | Jackman et al., (2018) | NSSI, self-injurious behaviour | Intentional direct harm to the body’s surface without intent to die, highlighting the emergence of self-harm as a distinct diagnosis in the DSM. Self-injurious behaviour is maintained by its regulation of an individual’s affective experience or social situation. The model describes risk factors for NSSI such as adverse childhood experiences and genetic predisposition, highlighting interpersonal and intrapersonal vulnerability factors, affecting one’s response to stressful situations | Cutting the skin using a knife or razor, typically on the forearms or legs, other examples given are burning, picking, or scraping the skin, or hitting oneself | Discusses stigma related to minority status and identity as well as proximal minority stress processes of concealment and expectations of rejection, as contributing to NSSI. |
| **19** | Lockwood et al., (2021) | Self-harm | Self-injury or self-poisoning regardless of the intention and motivation behind the act, recognising the dimensional structure of self-harmful behaviour. | Not explicitly discussed | Self-harm was typically a quick, impulsive act in the context of overwhelming emotion, underpinned by cognitive processing deficits. The dynamic tension between emotion-based impulsivity and controlled deliberation was articulated in the moments before self-harm. Impulsive responses were perceived as modifiable. |
| **20** | Lundwall et al., (2025) | NSSI | Cutting, starving, scratching, hitting and slapping, head banging, smacking | Not explicitly defined, but described as an emotional regulation strategy for women more than for men, related to escape or avoidance | Exploring themes of powerlessness, self-punishment for social mistakes, and difficulty displaying the ‘right’ emotions were common themes related to reasons why autistic women self-injured. |
| **21** | Miller et al., (2021) | Self-harm, inclusive of NSSI | NSSI is deliberately harming one’s body without any intent to end life. This delineation between suicidal and non-suicidal self-harm is controversial, with some considering the distinction valid, but others arguing that suicidal and non-suicidal self-harm should not be separated, given that intent is often unclear, and many people engage in both. | Not explicitly discussed | Self-harm is experienced as powerful mental and physical urges, sated only by self-harming, suggesting that self-harm could be considered a compulsive rather than impulsive disorder, representing a new perspective on the behaviour. This included considering self-harm as emotion regulation, an addictive urge, self-harm to survive, interpersonal triggers and relationships. |
| **22** | Morris et al., (2015) | Self-harm | Self-injurious behaviours that do not have suicidal intent. Emotional dysregulation is positioned as a motivating factor as well as self-punishment and a need to increase feelings of control, a means of manage either too much or too little emotion. Self-injury alleviates emotional states of high intensity and negative valence, including frustration and anxiety. | Burning, cutting, self-poisoning, hair pulling, head banging, inserting objects into the skin, eyes and ears. | Exploring meanings behind self-harm such as people with LE experiencing childhood trauma and abuse, that involved been seen and not heard, using self-harm as a release, to get rid of pain and hurt, with a vicious cycle developing where self-harm was helpful but then was accompanied by a range of less salutary effects, people with LE described feel different or distanced from the rest of society. |
| **23** | Mughal et al., (2023) | Self-harm | Self-injury or self-poisoning irrespective of suicidal intent. Risk factors include family discord, bullying, and mental health difficulties. Discusses Nock’s functional model, referring to intrapersonal and interpersonal reinforcement. | Self-poisoning, cutting, burning and pinching | Self-harm was used to handle emotional states, predominantly distress, and consisting of recognising emotions, expressing them, converting emotional pain into physical pain and avoiding and suppressing difficult thoughts. Self-harm was used as a self-punishment, because of a failure to meet individual expectations, and self-criticism, coping with mental illness and trauma. |
| **24** | Naz et al., (2021) | Self-harm | Self-harm is one of the strongest predicators of death by suicide in adolescence, self-harm includes negative emotions toward the self, lack of control over their lives and disturbed interpersonal relationships. | Not explicitly discussed | Interpersonal conflicts and emotional crisis were seen to predispose self-harm; self-harm being seen as the only option and the impact of self-harm. |
| **25** | Norman et al., (2022) | Self-harm | Intentional self-poisoning or self-injury irrespective of the apparent purpose of the act. Explore the differences across clinical and personal conceptualisations, where clinicians may see it negatively, but people with LE consider it a ‘necessary pain’. | Not explicitly discussed | People with LE were found to acknowledge and resist social constructs of self-harm as bad, exploring how self-harm works and then does not, and how it is a ‘symptom’ with which meaning lies beyond the behaviour, and how self-harm is part of people’s stories of their lives. |
| **26** | Norman et al., (2023) | Self-harm | Self-poisoning or self-injury, irrespective of motivation, explores how it is increasing amongst adolescent girls and young women. Self-harm can serve an interpersonal function, such as eliciting a response from others and/or an intrapersonal function, such as regulating overwhelming emotion. Self-harm tends to be preceded by increased negative affect and then followed by relief. | Not explicitly discussed | People with LE discuss self-harm as a way to prove oneself as real, associated with difficulties in understanding feelings, with discussions of a logical versus illogical self, with a tendency to describe two sides often in dialogue or conflict, others describe difficult understanding themselves as a disconnection from their physical bodies, with self-harm making people feel real. Others with LE describe self-harm as reliving the distress of disconnection, where failure and difficult describing feelings leads to disconnection, self-harm serves as a language and other ways of understanding one’s experience, through diagnosis and labelling or borrowing words, through song lyrics for example, helped. |
| **27** | Pollock et al., (2021) | Self-harm, parasuicide | Non-fatal act in which an individual deliberately initiates a non-habitual behaviour, that without intervention from others will cause self-harm or deliberately ingests a substance in excess of the prescribed or generally recognised therapeutic dosage, and which is aimed at realising changes that the person desires via the actual or expected physical consequences. | Overdose, cutting | Self-harm arose out of contextual factors, such as ACEs, poor relationships and difficulties in support networks, exposure to self-harm, through peers engaging or via social media, meanings behind self-harm was a need for self-punishment, its addictive qualities, and people with LE described concealment of harming behaviour, as well as seeing peers continuing to engage, people also described experiencing mental distress problems. Finally, people described how professional or peer support that helps or hinders reduction and cessation. |
| **28** | Rosenrot & Lewis (2020) | NSSI | Entails the deliberate damaging of body tissue in the absence of conscious suicidal intent | Cutting, burning | Barriers to disclosure, such as shame, concern about others, and disclosure recipients’ response included silence, avoidance and understanding were explored. Shame seemed a central part of disclosure of NSSI. |
| **29** | Russell et al., (2010) | Self-harm | Deliberate, self-initiated, and non-fatal act, carried out in the knowledge that it is potentially harmful, the deliberate, direct destruction or alteration of body tissue without conscious suicidal intent. Discusses an overview of the different themes to understand self-harm across the literature | Not explicitly discussed | People with LE describe soothing practices, dissociation and an ambivalent stuckness around separation versus incorporation, other meanings, such as existential vulnerability and openness, sacrifice, lack of boundaries and insatiability arose. |
| **30** | Sabo et al., (2025) | Self-harm | Self-harm is seen to be likely influenced by a range of individual and systemic factors. | Not explicitly discussed | Experiences of social isolation or rejection were perceived as risk, and social connection was seen as protective, overwhelming emotions can lead to self-harm and hinder help-seeking, finally misunderstood and inadequately accommodated by clinicians were directly impacted by the degree to which their clinician was perceived to be knowledgeable about autism. |
| **31** | Simpoulou & Chandler (2020) | Self-harm | Self-harm as a form of self-care, an attempt to provide meaning in the face of incoherence, emptiness or torment, seeing ‘providing meaning’ as part of an attempt at self-care. | Not explicitly discussed | Repetitive, permanent, deep, superficial, pleasing, ongoing, returning; it is an attempt at keeping oneself going even if it is by harming oneself. Sustaining self-harm,  sustains one’s sense of self. Self-harming becomes a personally meaningful experience – a provision of, or an attempt at, a meaning. This imagining provides an alternative vision of how self-harm might be thought as an act of care towards the self. Self-harm seen in this way, makes attempts at healing by making attempts at meaning through a relationship with one’s body. Drawing on the significance of the  early relationship with one’s own body as both a point of contact and differentiation between the self and world, we consider how the turn to the body in self-harming can be seen as a turn to a refuge through an  act of self-care. |
| **32** | Stanicke (2021) | Self-harm | Direct self-harm may or may not involve suicidal intent, referring to intentional self-poisoning or self-injury, irrespective of the type of motive or extent of suicidal intent, NSSI refers to deliberate destruction of one’s bodily tissue in the absence of suicidal intent and for reasons not socially sanctioned. | Not explicitly discussed | Self-harm speaks to a means of handling inner pain and vulnerability where young people describe feeling that they deserve self-harm, that feelings are experienced as not being wanted, including diffusive emotionality, feeling that something is wrong with the self, feeling harmed but that no one else cares. |
| **33** | Troya et al., (2019) | Self-harm | Any act of self-poisoning or self-injury, irrespective of motivation. Risk factors in older adults included physical illness, with loss of control, increased loneliness and perceived burdensome of aging were reported to be self-harm motivations. | Self-poisoning | Accumulated stressors over the life course led to older adults being vulnerable to self-harm. Stressors included adverse events, loss, interpersonal and health problems. A sense of shame and stigma amongst older people using self-harm to manage distress was reported. |
| **34** | Williams et al., (2023) | Self-harm thoughts and behaviours, self-injury | Self-injury or poisoning irrespective of suicidal intent, emphasised that they approached self-harm within a dimensional approach to include experiences with and without suicidal intent. | Genital self-mutilation, | Struggling with processing and understanding one’s own LGBTQ+ identity, negative responses to being LGBQT+ and life stressors. |
| **35** | Witcher et al., (2025) | (Repetitive) Self-harm | Intentional self-poisoning or injury, irrespective of the apparent purpose of the act, and as an expression of personal distress, not an illness, this does not imply motive or intent of the behaviour. In this study, methods such as overeating, body piercing, body tattooing, excessive consumption of alcohol or recreational drugs, restriction or starvation were excluded. Defines RSH as inclusive of five or more occurrences of self-harm within a 12-month period. | Cutting, burning, branding, scratching, picking at skin or reopening wounds, biting, head banging, hair pulling, hitting and bone breaking. | Describing self-harm as arising from a need to punish oneself, having the urge to self-harm, self-harm feeling addictive, experiencing conflicting relationship with self-harm and oneself, a cycle of self-harm where it had different functions and consequences, managing emotions, allowing oneself to function, caring for myself, controlling and feeling guilt and shame after self-harm, discussing responding to other’s reactions, breaking the self-harm cycle, and relapsing. |
| **36** | Woodley et al., (2021) | Self-harm, including self-mutilation, self-hurting and self-injury | Intentional injury of one’s own body tissue without suicidal intent, with increased suicidal risk and mental health difficulties. Acknowledges many different theoretical perspectives on self-harm, where the medical/diagnostic perspective is more dominant, that often involves a pathologising discourse, where self-harm is a problem behaviour that must be stopped. Psychological theories consider how self-harm can be connected to experiences of trauma, attachment and emergent self-identity. Sociocultural perspectives emphasise other meanings like healing oneself, restoring power and control, and the body as a means of conveying internal pain. | Cutting, burning, scratching, and interfering with wound healing. | People with LE discussed being involved in risk management of their self-harm, including managing the consequences of self-harm, exercising control in the process and an awareness of social context. This included people exploring that they did not like self-harm, but it works, highlighting how it helps, practical risk management, the impact of self-harm on others and times when self-harm became more dangerous. |
